# Targeted Isolation of Antibodies Directed against Major Sites of SIV Env Vulnerability

**DOI:** 10.1371/journal.ppat.1005537

**Published:** 2016-04-11

**Authors:** Rosemarie D. Mason, Hugh C. Welles, Cameron Adams, Bimal K. Chakrabarti, Jason Gorman, Tongqing Zhou, Richard Nguyen, Sijy O’Dell, Sabrina Lusvarghi, Carole A. Bewley, Hui Li, George M. Shaw, Zizhang Sheng, Lawrence Shapiro, Richard Wyatt, Peter D. Kwong, John R. Mascola, Mario Roederer

**Affiliations:** 1 Vaccine Research Center, National Institute of Allergy and Infectious Diseases (NIAID), National Institutes of Health (NIH), Bethesda, Maryland, United States of America; 2 International AIDS Vaccine Initiative (IAVI) HIV Vaccine Design Program, Translational Health Science and Technology Institute, Haryana, India; 3 Laboratory of Bioorganic Chemistry, National Institute of Diabetes and Digestive and Kidney Diseases (NIDDK), National Institutes of Health (NIH), Bethesda, Maryland, United States of America; 4 Departments of Medicine and Microbiology, Perelman School of Medicine, University of Pennsylvania, Philadelphia, Pennsylvania, United States of America; 5 Department of Biochemistry and Molecular Biophysics and Department of Systems Biology, Columbia University, New York, New York, United States of America; 6 IAVI Neutralizing Antibody Center, Department of Immunology and Microbial Science, The Scripps Research Institute, La Jolla, California, United States of America; University of Zurich, SWITZERLAND

## Abstract

The simian immunodeficiency virus (SIV) challenge model of lentiviral infection is often used as a model to human immunodeficiency virus type 1 (HIV-1) for studying vaccine mediated and immune correlates of protection. However, knowledge of the structure of the SIV envelope (Env) glycoprotein is limited, as is knowledge of binding specificity, function and potential efficacy of SIV antibody responses. In this study we describe the use of a competitive probe binding sort strategy as well as scaffolded probes for targeted isolation of SIV Env-specific monoclonal antibodies (mAbs). We isolated nearly 70 SIV-specific mAbs directed against major sites of SIV Env vulnerability analogous to broadly neutralizing antibody (bnAb) targets of HIV-1, namely, the CD4 binding site (CD4bs), CD4-induced (CD4i)-site, peptide epitopes in variable loops 1, 2 and 3 (V1, V2, V3) and potentially glycan targets of SIV Env. The range of SIV mAbs isolated includes those exhibiting varying degrees of neutralization breadth and potency as well as others that demonstrated binding but not neutralization. Several SIV mAbs displayed broad and potent neutralization of a diverse panel of 20 SIV viral isolates with some also neutralizing HIV-2_7312A_. This extensive panel of SIV mAbs will facilitate more effective use of the SIV non-human primate (NHP) model for understanding the variables in development of a HIV vaccine or immunotherapy.

## Author Summary

An antibody-based approach targeting human immunodeficiency virus (HIV) envelope (Env) protein may eventually prove to be effective in treating or preventing HIV infection. However, before any candidate HIV treatment or vaccine can be tested in humans, it must first be evaluated in nonhuman primates (NHPs)–the closest living relatives to humans. Simian immunodeficiency virus (SIV) is the closest available non-chimeric virus—NHP model for studying and testing HIV vaccines or therapies. The SIV model complements the simian-human immunodeficiency virus (SHIV) model in distinctive ways, although less is known about SIV Env-specific antibody responses in NHPs. There are several sites on HIV Env that are vulnerable to antibody-mediated protection, and here we isolated and analyzed monoclonal antibodies (mAbs) from NHPs targeting analogous sites on SIV Env. In particular, we studied mAbs for their ability to bind the viral Env protein and to block infection of cells by widely divergent strains of SIV. These well-characterized SIV Env-specific antibodies will allow for more thorough NHP pre-clinical testing of various antibody-based SIV/HIV vaccine and immunotherapeutic strategies before proceeding to human clinical trials and may yield unanticipated findings relating to molecular mechanisms underlying the unusual breadth of neutralization observed in HIV-2 infection.

## Introduction

Generating protective antibody responses by vaccination is the ultimate goal of an effective HIV vaccine [1–4]. As such, a number of highly potent bnAbs targeting major sites of HIV-1 Env vulnerability such as the CD4bs [5–8], peptido-glycans of variable loops V1, V2 and V3 [9–12], the membrane-proximal external region (MPER) [13–15] and the gp41-gp120 interface [16, 17] have been isolated and examined for their potential impact on HIV vaccine design [18–20]. The specificity and effector functions of protective, non-neutralizing antibodies (pnnAbs) are likewise being scrutinized for their potential complementary role toward protection against HIV infection [21–24]. However, recent studies highlight the challenges to developing an effective HIV-1 vaccine [25–34] and suggest that a better understanding of SIV Env-specific antibody responses might complement and inform HIV vaccine design. This possibility is underscored by the protective effects of Env targeted antibodies elicited by adenovirus-vectored immunogens in SIV protection trials [35–38] and the surprising discovery that HIV-2, a derivative of SIVsmm, commonly elicits bNabs in natural human infection [39–41]. A better understanding of protective SIV Env-specific antibody responses may thus facilitate more effective use of the SIV challenge model to evaluate candidate vaccines and immunotherapies before proceeding to costly, time consuming and resource intensive human clinical trials.

Design of a HIV immunogen that can i) focus the antibody response to protective yet subdominant or sterically hindered epitopes, ii) engage Abs encoded by germline B cell receptors (BCRs) and iii) drive sufficient antibody affinity maturation to generate protective antibody responses will likely require iterative immunogen design [42]. Additional work will be required to optimize the antibody specificities and functions, alone or in combination, which are necessary and sufficient to protect against HIV infection. Finally, it will be necessary to assess which vaccine regimens and adjuvant combinations can achieve the desired germline BCR engagement, affinity maturation, antibody persistence and ultimately, protective efficacy against HIV challenge. All of these unanswered questions necessitate a relevant NHP model for HIV vaccine research

The SHIV—NHP model of HIV infection has been used extensively to study antibody-mediated correlates of protection [43–46]. Chimeric SHIVs are often constructed by replacing the envelope gene and additional accessory proteins of the pathogenic molecular clone of SIVmac239 with corresponding genes from selected HIV-1 subtypes followed by *in vivo* passaging for enhanced virus replication [47]. Such constructs have proven invaluable for screening candidate HIV immunogens and the development of pathogenic SHIV chimeras has allowed for testing of antibody-mediated protection [48–53]. However, SHIVs have limited genetic diversity [54, 55] compared with SIV challenge stocks that reflect the diversity present in primary circulating isolates of HIV-1 [56]. Thus, protection against SIV may better estimate the protective efficacy of a HIV vaccine and may complement the SHIV model used with clinically relevant reagents. Indeed, vaccine protection against acquisition of neutralization resistant SIV challenges in rhesus macaques suggests a role for antibody-mediated protection [35–37, 57]. However, the epitope specificities and effector functions of SIV-specific antibodies mediating protection have yet to be fully characterized. Thus, developing reagents to study SIV-specific antibody responses in NHP can provide an informative model for defining antibody-mediated correlates of protection.

Our overall goal was to identify SIV-specific antibodies from macaques that may inform the development of effective HIV antibody-based interventions. Some of the most potent HIV-1 bnAbs target the CD4bs, variable regions V1/V2 and the glycan/V3 loop of gp120 [1]. Given the paucity of SIV-specific probes, we designed scaffolded probes to isolate SIV V1V2-specific mAbs and developed a novel competitive probe binding procedure for isolation of SIV mAbs targeting the CD4bs as well as high-mannose glycans on gp120. Both the scaffolded probes and competitive probe binding technique were highly efficient for the targeted isolation of SIV-specific B cells. Subsequent cloning, expression and characterization of individual mAbs identified many novel, potent mAbs targeting multiple sites of SIV Env vulnerability, including the first reported SIV CD4bs-specific neutralizing mAbs isolated from SIV-infected rhesus macaques.

## Materials and Methods

### Indian-origin rhesus macaque specimens

SIV-positive plasma and peripheral blood mononuclear cells (PBMC) were obtained from previously completed animal study protocols [35, 38, 58] (S1 Table).

### Plasmids

A protein scaffold (1JO8) [59] that provides an appropriate hairpin was identified to suitably incorporate the SIV Env V1V2 region based on stable expression, clash score and solvent accessibility. This scaffold allows V1V2 to be expressed at high yield in a context that maintains proper conformation of a native V1V2 protomer [60]. A soluble trimeric SIVmac239 gp140 foldon protein expression vector was generated by encoding SIVmac239 from residues 1 thru 722, followed by the foldon trimerization motif as previously described [61]. The following mammalian expression vectors were used for synthesis of SIV proteins: pcDNA3.1(-) encoding SIVmac239 gp140 foldon trimer (FT) and pVRC8400 [62] encoding either 1JO8-scaffolded SIVsmE660.CP3C or SIVsmE660.CR54 V1V2 loop sequences (GenScript). All constructs contained C-terminus 6X His-tag for protein purification followed by an Avi-tag motif for biotinylation. The SIVmac239 ΔV1V2V3 gp120 plasmid encoding gp120 residues 44 to 492 (HXBc2 numbering) with truncations in the V1V2 and V3 regions as in previously reported HIV-1 CoreE gp120 proteins [63], in which residues 124 to 198 in the V1V2 loop and residues 302 to 323 in the V3 loop of SIV gp120 were replaced with GG and GGSGSG linkers, respectively and kindly provided by Andrés Finzi. Construction of a synthetic gene encoding full-length cyanovirin-N (CVN) inserted into a pET-26(+) vector (Novagen) has been previously described [64]. The CD4-Ig plasmid encoding the first two N-terminal domains of the CD4 molecule which are sufficient for high-affinity gp120 binding fused with the Fc region of human IgG1 was kindly provided by Joseph Sodroski [65].

### Protein production and purification

All SIV proteins and CD4-Ig were expressed by transient transfection of 293Freestyle (293F) cells in serum-free medium using 293fectin transfection reagent (Invitrogen) according to manufacturer’s instructions. Cell culture supernatants were harvested 6 days post-transfection, passed through a 0.22 μm filter to remove any cell debris and supplemented with protease inhibitor tablets (Roche). All SIV proteins were purified using Ni Sepharose excel affinity media (GE Healthcare) followed by size exclusion chromatography (SEC) on a HiLoad 16/600 200 pg Superdex column (GE Healthcare). CD4-Ig was purified using a recombinant protein A affinity column (GE Healthcare) as previously described [66]. Recombinant CVN was produced as previously reported [67]. Briefly, CVN was expressed in the BL21-DE3 *E*. *coli* strain (New England Biolabs), followed by purification using reversed-phase chromatograpy (Sep-Pak Vac 35cc (10g) tC18 cartridges, Waters) and gel-filtration (Superdex 75, GE Healthcare) to ensure separation of monomeric and domain-swapped dimeric CVN.

### Targeted isolation of SIV-specific B cells by fluorescence activated cell sorting (FACS)

Cryopreserved PBMC were thawed and stained with LIVE/DEAD Fixable Violet Dead Cell Stain (Life Technologies) as previously described [68, 69]. Cells were washed and stained with an antibody cocktail of CD3 (clone SP34-2, BD Biosciences), CD4 (clone OKT4, BioLegend), CD8 (clone RPA-T8, BioLegend), CD14 (clone M5E2, BioLegend), CD20 (clone 2H7, BioLegend), IgG (G18-145, BD Biosciences) and IgM (clone G20-127, BD Biosciences) at room temperature in the dark for 20 mins. The cells were washed twice with PBS and subsequently stained with fluorescently labeled SIV probes to stain for CD4bs-, cyanovirin binding site (CVNbs)- or V1V2-specific B cells.

For staining of CD4bs-and CVNbs-specific B cells, SIVmac239 gp140 FT was used in combination with 4-fold or 5-fold molar excess of CD4-Ig fusion protein or CVN protein, respectively. Cells were first re-suspended in 200 μl PBS with CD4-Ig:SIVmac239 gp140-PE or CVN:SIVmac239 gp140-PE, respectively, incubated at room temperature in the dark for 20 mins followed by 3 washes with PBS and then re-suspended in 200 μl PBS containing SIVgp140-APC and incubated further for 20 mins at room temperature in the dark. For staining of V1V2-specific B cells, cells were re-suspended in 200 μl PBS containing PE-labeled 1JO8 SIVsmE660.CP3C V1V2 and/or 1JO8 SIVsmE660.CR54 V1V2 and incubated for 20 mins at room temperature in the dark. The stained cells were washed 3 times and re-suspended in 1 ml of PBS, passed through a 70 μm cell mesh (BD Biosciences) then analyzed and sorted with a modified 3-laser FACSAria cell sorter using the FACSDiva software (BD Biosciences). Probe-positive B cells were sorted as single cells into wells of a 96-well plate containing lysis solution as previously described [5]. Flow cytometric data was subsequently analyzed using FlowJo (v9.7.5).

### RT-PCR, cloning and expression of immunoglobulin genes

Single B cell RNA was reverse transcribed as previously described [5], diluted 2-fold by addition of 26 μl nuclease-free water and the cDNA plates were stored at -20°C. Individual rhesus immunoglobulin (Ig) heavy (H), light kappa (Lκ) and light lambda (Lλ) chain genes were amplified by nested PCR using 5 μl cDNA as template. All PCR reactions were performed in 96-well PCR plates in a total volume of 50 μl. For first round amplification, first-round rhesus-specific PCR primers (S2–S4 Tables) were used to amplify gene transcripts containing 2 U of HotStar Taq Plus DNA Polymerase (QIAGEN), 1 μl dNTP-Mix (10 mM each nucleotide) (QIAGEN), 0.5 μg carrier RNA, 1 mM MgCl2, 1 μl forward primer mix (50 μM each primer), 1 μl reverse primer (25 μM each primer), using the following PCR program: 5 min at 94°C; 50 cycles of 30 sec at 94°C, 45 sec at 50°C, 45 sec at 72°C: followed by 10 min at 72°C. One-twentieth the volume of first-round PCR product was amplified by nested PCR with second-round rhesus-specific PCR primers (S2–S4 Tables) under the same conditions used for first round PCR. The second round of PCR was performed for 5 min at 94°C followed by 30 cycles of 30 sec at 94°C, 45 sec at 60°C, 45 sec at 72°C and a final 10 min extension at 72°C. Amplified PCR products were analyzed on 2% agarose gels (Embi-Tec) and positive reactions sequenced directly. PCR products with productive Igγ and IgLκ or IgLλ sequence were re-amplified with 3 μl of unpurified first round PCR product as template and combinations of single gene-specific V and J gene primers incorporating unique restriction digest sites. Resulting PCR products were run on a 1% agarose gel and purified with QIAGEN Gel Extraction Kit (QIAGEN) and eluted with 25 μl nuclease-free water (Quality Biolgical). Purified PCR products were digested with appropriate restriction digest enzymes AgeI, Nhel, BsiWI and ScaI (all from ThermoScientific) before ligation into rhesus Igγ, IgLκ and IgLλ expression vectors containing a murine Ig gene signal peptide sequence (GenBank accession number DQ407610) and a multiple cloning site upstream of the rhesus Igγ, Igκ or Igλ constant regions (all 3 expression vectors were kindly provided by Kevin Saunders). Transcription of these expression vectors is under the influence of human cytomegalovirus (HCMV) promoter allowing clones to be selected based on resistance to kanamycin. Full-length IgG was expressed as previously described [5] by co-transfecting 293F cells with equal amounts of paired heavy and light chain plasmids then purified using Protein A Sepharose beads (GE Healthcare) according to manufacturer’s instructions.

#### Immunoglobulin gene family analysis

The IgG heavy chain nucleotide sequences were assigned to a germline variable gene using local implemented IgBlast (http://www.ncbi.nlm.nih.gov/igblast/). A new germline V gene database was used for heavy chain germline gene assignment and somatic mutation calculation [70]. Antibody light chain sequences were compared to the rhesus monkey immunoglobulin germline sequences using IMGT/V-QUEST from IMGT [71, 72]. Each antibody heavy chain sequence and the assigned germline V gene were aligned using Muscle and then the nucleotide (nt) divergence was calculated. The heavy-chain complementary determining region 3 (CDR3) sequence was extracted using the conserved C at the end of V region and WGXG motif for heavy chain (X represents any of the 20 amino acids) and the conserved C at the end of V region and FGXG motifs for kappa and lambda chains. All extracted CDR3 regions were under manual inspection.

### Protein and peptide binding and competition assays

Binding of SIV-specific mAbs to purified proteins or synthetic peptides was measured by enzyme-linked immunosorbent assay (ELISA) as previously described [5]. For CD4bs competition ELISA, plates were coated with 2 μg/ml SIVmac239 gp140 FT in PBS at 4°C overnight. After blocking with 200 μl B3T buffer (150mM NaCl, 50mM Tris-HCl, 1mM EDTA, 3.3% fetal bovine serum, 2% bovine albumin, 0.07% Tween-20), serial dilutions of unlabeled competitor mAbs were added to captured SIVmac239 gp140 FT in 100 μl B3T buffer for 15 mins prior to addition of biotinylated CD4-Ig (at a concentration determined to yield O.D. of roughly 1.0–2.0). Alternatively, competition with sCD4 was performed by addition titrating amounts of sCD4 to SIVmac239 gp140 FT-coated plates for 15 mins prior to addition of individual mAbs (at a concentration determined to yield O.D. of roughly 1.0–2.0) and binding was detected by HRP-conjugated anti-monkey IgG (Rockland Immunochemicals) at a 1:5,000 dilution for 1 hour. Antibody cross-competition ELISA was performed by adding titrations of unlabeled competitor mAbs to SIVmac239 gp140 FT-coated plates for 15 mins prior to addition of individual biotinylated mAbs (at a concentration determined to yield O.D. of roughly 1.0–2.0). Plates were incubated for 1 hr at 37°C, washed 3 times with B3T buffer followed by incubation with streptavidin-horseradish peroxidase (HRP) for 1 hr at 37°C. For peptide competition ELISA, titrations of peptides were added to SIVmac239 gp140 FT-coated plates for 15 mins prior to addition of individual mAbs (at a concentration determined to yield O.D. of roughly 1.0–2.0) and binding detected by HRP-conjugated anti-monkey IgG (Rockland Immunochemicals) as above. The signal was developed by addition of 3,3′,5,5′-tetramethylbenzidine (TMB) substrate (SureBlue; KPL) for 10 min. Reactions were terminated with 1 N sulfuric acid, and the optical density (OD) was read at 450 nm. The following reagent was obtained through the NIH AIDS Reagent Program, Division of AIDS, NIAID, NIH: SIVmac239 Env Peptide Set. The following reagent was obtained through the NIH AIDS Reagent Program, Division of AIDS, NIAID, NIH: Soluble Human CD4 from Progenics and sCD4-183 from Pharmacia, Inc. [73]

### Viral neutralization and competition assays

Plasmid DNA encoding SIV gp160 was used in combination with a luciferase reporter plasmid containing the essential HIV structural genes to produce SIV Env pseudoviruses as described previously [74]. Plasmids encoding SIV gp160 for clones SIVsmE660.11 [75], SIVmac239.cs.23 [76], SIVmac251.6 [76] and SIVmac251.cs.41 [77] were generously provided by David Montefiori. Plasmids encoding SIV gp160 for clones SIVsm (FFv 18Nov04 ENVPL2.1, FJv 15Nov06 ENVPL2.1, FWk 12Aug04 ENVPL4.1 and RSo8 17Jan06 ENVPL1.1) and SIVmac251 (RSo8 17Jan06 ENVPL1.1 and RZj5 9Apr09 ENVPL2.1) were kindly provided by Cynthia Derdeyn [78]. Full-length infectious molecular clones of transmitted/founder viruses corresponding to SIVsmm lineage 1 (RM174.V1,V2,V3.tf), 5 (FTq) and “outlier” (SL92b) were derived by methods previously described [79]. In brief, naïve Indian rhesus macaques were inoculated intravenously with plasma from sooty mangabey monkeys naturally infected with the SIVsmm lineage 1 or 5 viruses or with a primary lymphocyte culture of SIVsmm SL92b obtained from a naturally infected sooty mangabey. All three rhesus macaques became productively and chronically infected. Chronic plasma from these three animals was then inoculated intravenously into three naïve Indian rhesus macaques. Twelve days later, acute infection plasma was collected, plasma viral RNA isolated, viral cDNA generated, full-length T/F SIVsmm sequences single genome amplified by limiting dilution PCR, and the products molecularly cloned, as described [79]. IMC sequences (GenBank accession numbers KU182919-23) were identical to the respective inferred T/F viral genomes, which represent examples of highly diverse naturally-occurring strains of SIVsmm [80]. Virus neutralization was measured using single round infection of TZM-bl target cells by SIV Env-pseudovirus or replication-competent viruses i.e. infectious molecular clones (IMC) in the presence of the protease inhibitor indinavir as previously described [81]. The 50% inhibitory concentration (IC_50_) was defined as the antibody concentration that caused a 50% reduction in relative light units (RLU) compared to virus control wells after subtraction of background RLU. Half-maximal inhibitory concentration (HalfMax) was defined as the antibody concentration that caused a 50% reduction in maximum neutralization for a given mAb while the maximum neutralization (VMax) was defined as the maximum % neutralization observed over the range of mAb concentrations tested.

## Results

### Isolation of rhesus antibodies targeting SIV CD4bs by competitive probe binding

**Fig 1:**
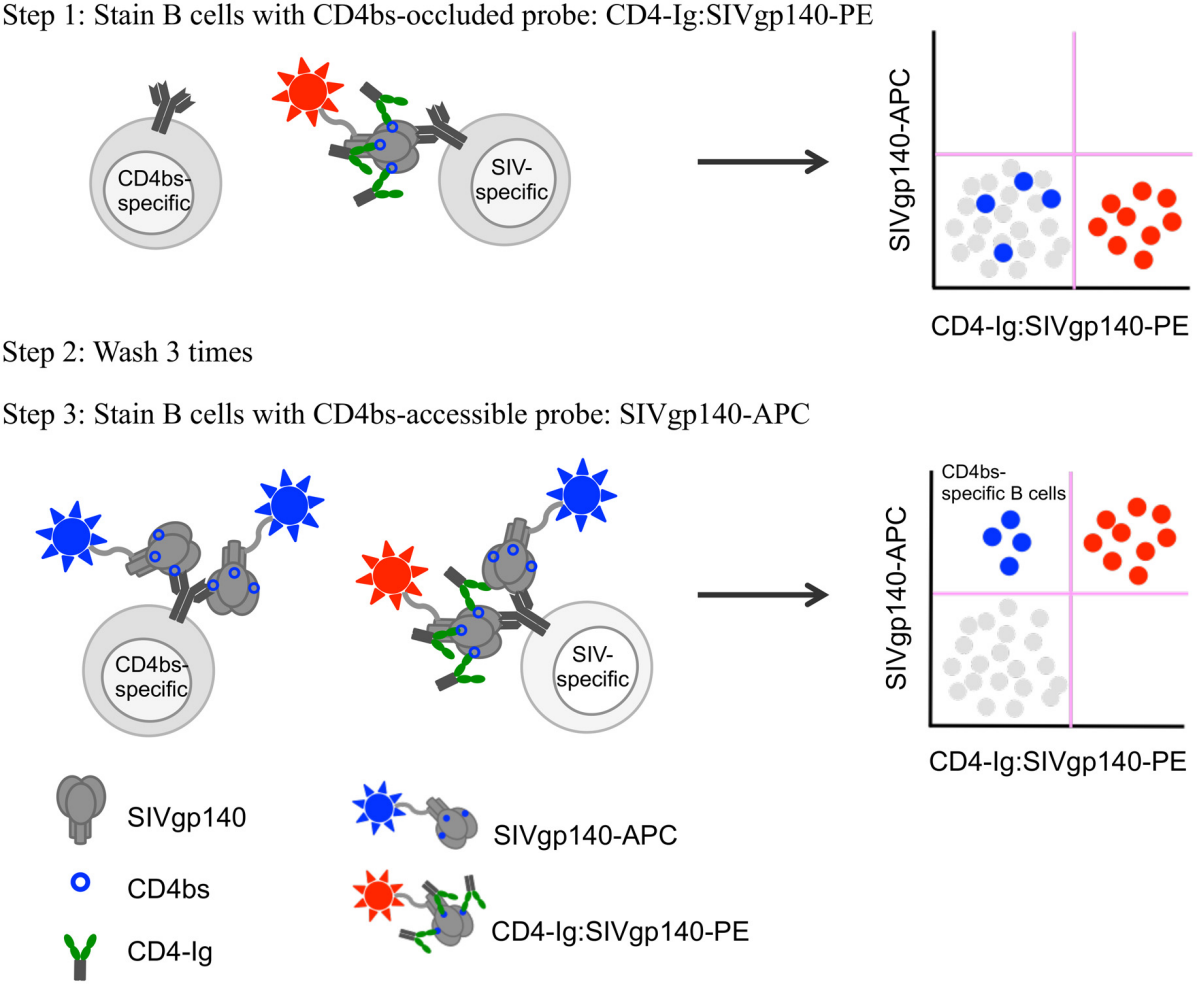
Targeted isolation of SIV CD4bs-specific B cells. Schematic illustrating competitive probe binding strategy for targeted isolation of CD4bs-specific B cells. Cells are first stained with a CD4bs-occluded probe (i.e. CD4-Ig:SIVgp140-PE) to label all SIV-specific B cells (red) except those that are specific for the CD4bs (blue) (Step 1) as shown in FACS plot (top right). After extensive washing to remove unbound CD4-Ig:SIVgp140-PE from first stain (Step 1), cells are stained with a CD4bs-accessible probe (i.e. SIVgp140-APC in the absence of any competing CD4-Ig) (Step 3). All SIV-specific B cells, including those specific for the CD4bs, will bind to this probe with putative CD4bs-specific B cells (blue) being positive for SIVgp140-APC and negative for CD4-Ig:SIVgp140-PE as shown in FACS plot (bottom right).

To identify SIV CD4bs-specific B cells, we prepared 2 probes exhibiting differential binding capacity for CD4bs-specific B cells (Fig 1). A CD4bs-occluded SIVmac239 gp140 FT probe was prepared by mixing PE-conjugated SIVmac239 gp140 FT with a 4-fold molar excess of CD4-Ig fusion protein [66]. An APC-conjugated SIVmac239 gp140 FT served as a CD4bs-accessible SIVmac239 gp140 probe. SIV CD4bs-specific B cells were identified by first staining cells with CD4-Ig:SIVmac239 gp140-PE to label all SIV-specific B cells except those blocked by excess CD4-Ig (Fig 1). After extensive washing to remove unbound CD4-Ig:SIVmac239 gp140-PE and excess CD4-Ig, cells were stained with SIVmac239 gp140-APC in order to label all SIV-specific (including CD4bs-specific) B cells (Fig 1). Thus, cells stained negative for CD4-Ig:SIVmac239 gp140-PE but positive for SIVmac239 gp140-APC identified putative CD4bs-specific B cells. B cells binding to both CD4-Ig:SIVmac239 gp140-PE and SIVmac239 gp140-APC would be expected to bind to gp140 at epitopes outside of the CD4bs.

**Fig 2:**
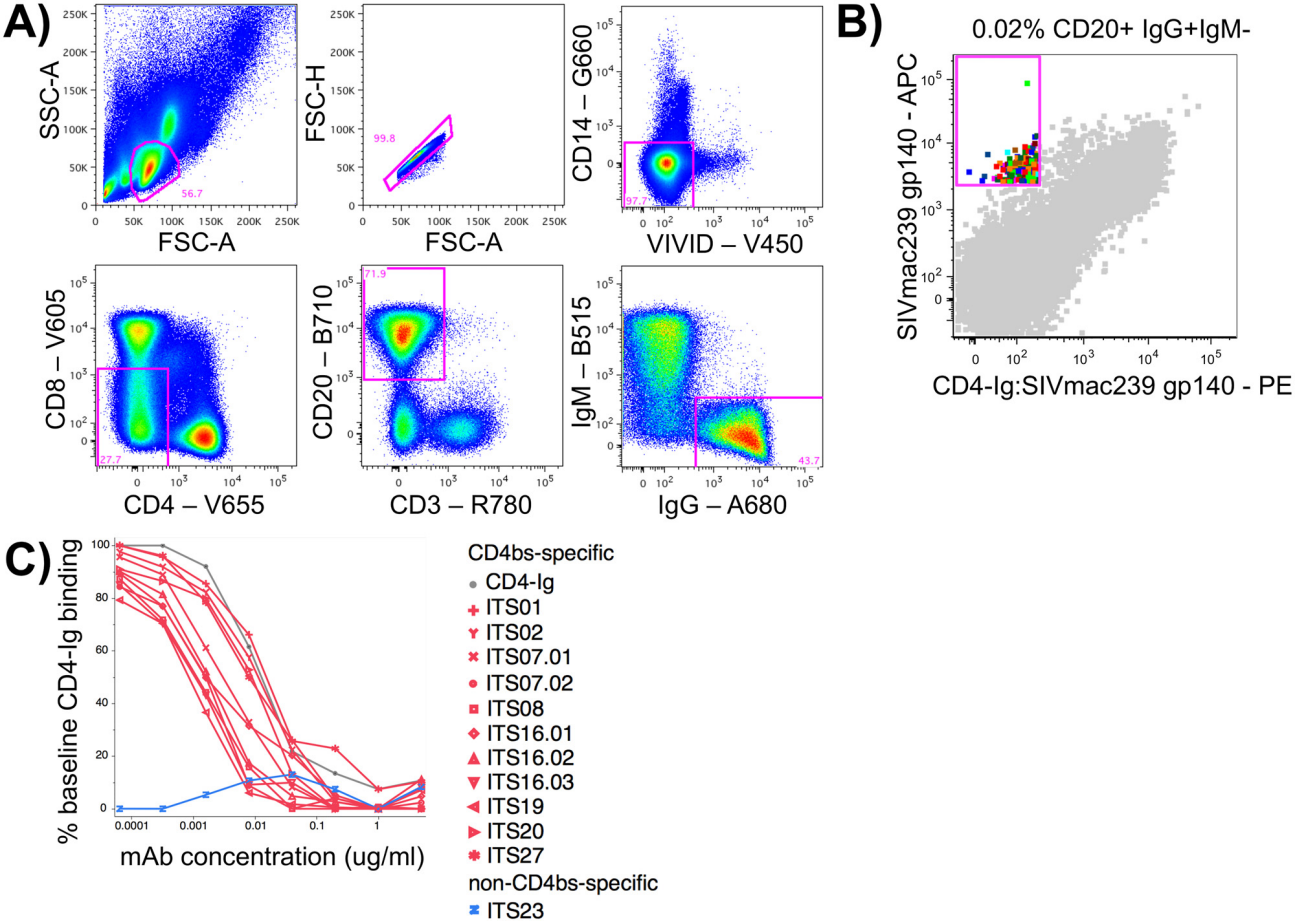
Isolation of CD4bs-specific B cells. A) Gating strategy for isolating rhesus macaque memory B cells i.e. lymphocytes / singlets / Live / CD3-CD4^-^CD8^-^CD14^-^ / CD20^+^ / IgM^-^ / IgG^+^ and B) FACS data overlay of individually sorted CD4bs-specific B cells (multicolored) as a percentage of total memory B cells (gray). C) Competition ELISA of biotinylated CD4-Ig binding to plate-bound SIVmac239 gp140 FT in the presence of individual competing mAbs graphed as percent inhibition = (OD without competitor—OD with competitor)/ OD without competitor X100. D) ELISA of non-binding mAbs at 5 μg/ml to plate-bound SIVmac239 gp140 FT in the presence of varying concentrations of sCD4.

In combination with a rhesus B cell staining panel (Fig 2A) we used this competitive probe binding staining procedure to sort 160 putative CD4bs-specific B cells from 4.4 million PBMC (0.02% of total B cells) from a SIVmac251-infected rhesus macaque (DBM5) [58] (S1 Table; Fig 2B). Amplification of immunoglobulin heavy and light chain variable regions yielded 37 matched heavy and light chain pairs belonging to 13 clonal families. We cloned and expressed 16 mAbs to characterize their binding and neutralization activity (Table 1). Among these 16 mAbs were 3 distinct clonal families including ITS07 and ITS16 mAbs. Rhesus heavy chain V gene usage of cloned mAbs was mostly restricted to IGHV4 alleles although ITS07 mAbs were IGHV3. Mutation frequency of heavy and light chain genes within the V region (based on nt sequence divergence from nearest assigned germline sequence) ranged from 2–11%.

**Table 1:** Genetic characteristics of mAbs isolated from animal DBM5 by CD4bs competition B cell sort. Clonally related mAbs share mAb ID numbers (i.e. ITS07) with additional numbering to denote individual clonal members (i.e. ITS07.01 and ITS07.02)

mAb ID	Rhesus heavy chain V gene	% Divergence (nt) from germline	CDRH3	Rhesus light chain V gene	% Divergence (nt) from germline	CDRL3
ITS01	IGHV4B*02	7%	ARGGNIWTGYHSTYFYY	IGLV1-14*01	6%	QSFDSSVSVQV
ITS02	IGHV4C*01	2%	ARDRSSYYTRGLDS	IGLV3-1*01	1%	QVWDSSSDHYI
ITS07.01	IGHV3M*01	10%	VRDGALDCTGSGCWVFDY	IGLV5-14*01	5%	AIGHSRGYT
ITS07.02	IGHV3M*01	10%	VRDGALDCTGSSCWVFDY	IGLV5-14*01	4%	AIGHSRGYT
ITS08	IGHV4G*02	10%	ARTWGIFGLVKNMRFDV	IGKV2S17*01	2%	MQGLEFPLT
ITS16.01	IGHV4L*02	5%	ARHSAGITAAWIGGNRRKTDY	IGKV1-12*01	3%	QQYNSDPHS
ITS16.02	IGHV4L*02	6%	ARHSAGLTAAWIGGRRRKTDY	IGKV1-12*01	3%	QQYTSDPHS
ITS16.03	IGHV4L*02	6%	ARHSAGLTAAWIGGRRRKTDY	IGKV1-12*01	4%	QQYNSDPHS
ITS19	IGHV4L*03	6%	ARHSAGLTAAWIGGSRRKTDY	IGKV1-14*01	2%	QQRNGYPWT
ITS20	IGHV4L*02	7%	ARHSTGLTAAWIGGRRRKIDY	IGKV3-5*01	6%	QETSDLFT
ITS23	IGHV4F*02	11%	ARDETKFGLVVS	IGLV1-7*01	5%	QSYDTNLRIL
ITS27	IGHV4G*01	11%	ARGSNIWTSYYDNWFDV	IGLV1S1*01	8%	STWDSSLSTGL
DBM5.2E10	IGHV4K*01	6%	ASDLLDFWTGYYTGWFDV	IGKV1-14*01	3%	QQRNGYPWT
DBM5.2E11	IGHV4K*01	6%	ASDLLDFWTGYYTGWFDV	IGKV1-12*01	4%	QQYNSDPHS
DBM5.2B3	IGHV4K*01	6%	ASDLLDFWTGYYTGWFDV	IGKV2S17*01	2%	MQGLEFPLT
DBM5.1A11	IGHV7-B*01	10%	ARRGFYWSDRGLDS	IGKV3S7*01	9%	QQTGDWPLS

### Binding and neutralizing activity of SIV CD4bs-specific mAbs

**Fig 3:**
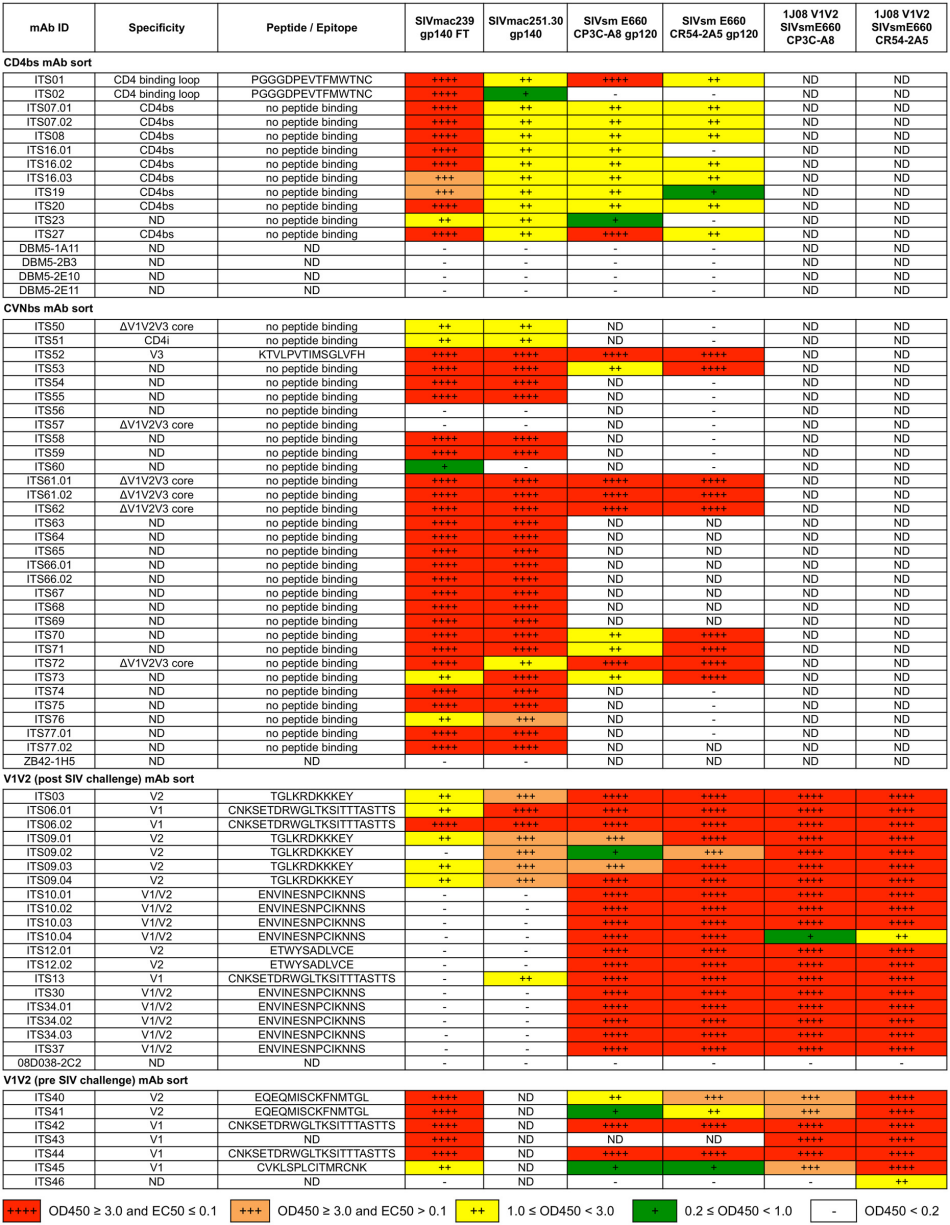
ELISA binding profiles of SIV-specific mAbs. mAbs cloned from individually sorted SIV Env-specific B cells were evaluated for binding to selected SIV Env proteins. ELISA binding was categorized as indicated by the legend. ND = not determined.

All putative CD4bs-specific mAbs were tested by ELISA for binding to SIVmac239 gp140 FT used for cell sorting. While 4 mAbs showed no detectable binding, 12 mAbs bound to SIVmac239 gp140 FT with varying affinities (Fig 3). These 12 mAbs also bound to monomeric SIVmac251.30 gp140 and in most cases to SIVsmE660.CP3C and/or smE660.CR54 gp120s as well. To evaluate whether mAbs cloned from B cells isolated using our 2-step competitive probe binding staining protocol were indeed specific for the CD4bs, we re-evaluated binding of the 12 SIV-specific mAbs to SIVmac239 gp140 FT by competition ELISA with CD4-Ig. Binding of CD4-Ig to SIVmac239 gp140 FT was effectively competed by 11 out of 12 mAbs (Fig 2C) confirming their specificity for the CD4bs.

For those mAbs that failed to bind to SIVmac239 gp140 FT, we hypothesized that residual CD4-Ig in the staining protocol may have facilitated SIVgp140-APC labeling of CD4-induced (CD4i)-specific B cells, i.e. binding to an epitope at or near the host cell co-receptor binding site which is exposed following binding of the primary receptor CD4. We tested whether the presence of soluble CD4 (sCD4) could facilitate binding of mAbs DBM5-2E10, 2E11, 2B3 and 1A11 to SIVmac239 gp140 FT. The addition of sCD4 had no effect on binding of these mAbs to SIVmac239 gp140 FT indicating these mAbs bound neither CD4bs- nor CD4i-specific B cells (Fig 2D). Nonetheless, our sort strategy was highly efficient for isolating CD4bs-specific B cells, as 11 out of 12 SIV-binding mAbs were CD4bs-specific.

**Fig 4:**
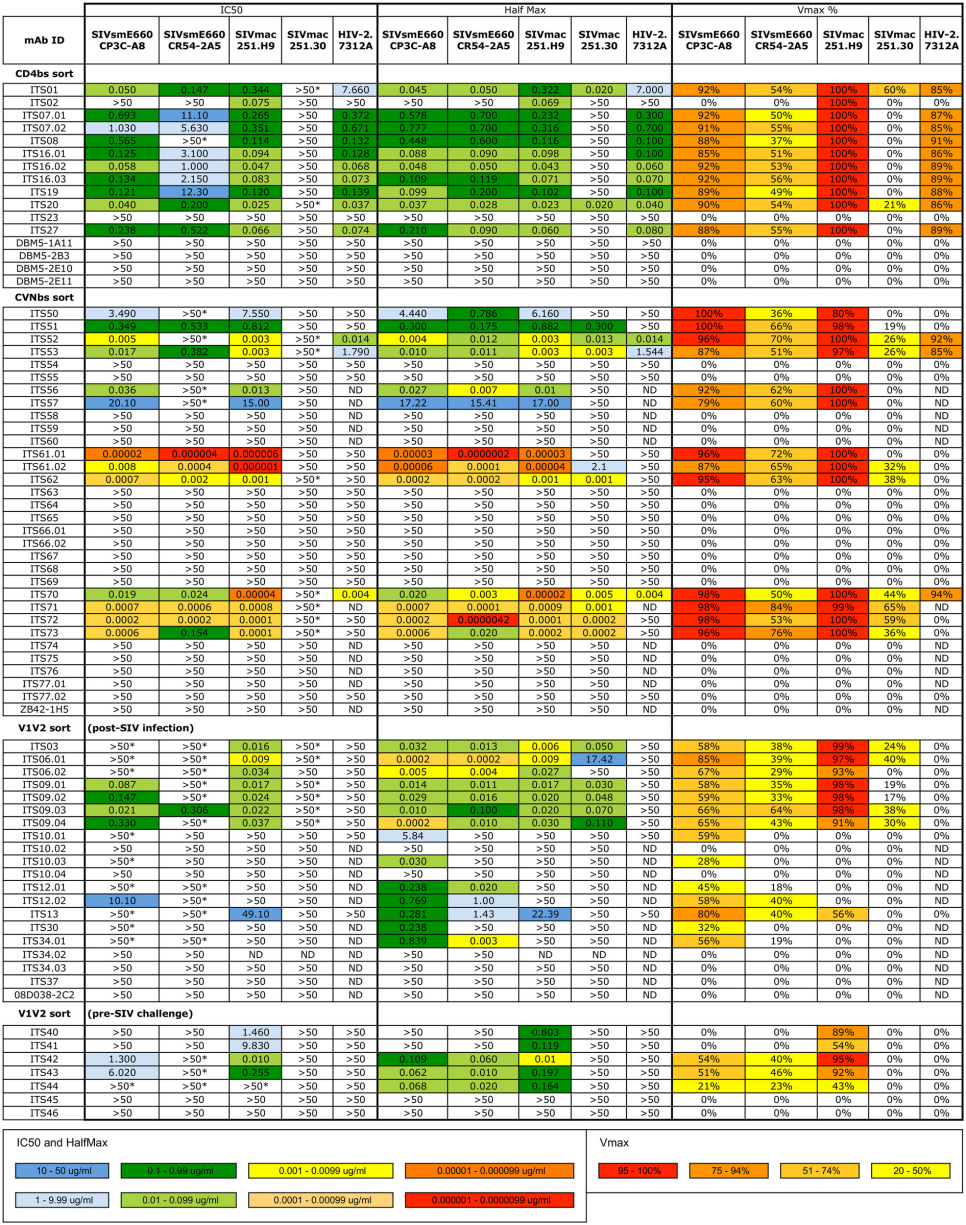
Summary of neutralization potency and cross-reactivity of SIV-specific mAbs. SIV mAbs cloned from targeted B cell sorts to isolate CD4bs-, CVNbs-and V1V2-specific B cells. Individually sorted SIV CD4bs-, CVNbs- and pre- and post-SIV infection V1V2-specific B cells were evaluated for neutralization of SIV pseudoviruses and HIV-2_7312A_. Values >50 indicate no neutralization while >50 with asterisk (*) indicates neutralization curve plateaued below 50%. ND = not determined.

We next assessed neutralizing activity of the 11 CD4bs-specific mAbs by TZM-bl assay against 4 SIV Env pseudoviruses and the IMC HIV-2_7312A_. All but one CD4bs-specific mAbs neutralized the highly neutralization-sensitive (tier 1) isolates SIVsmE660.CP3C and SIVmac251.H9.15, the moderately neutralization-resistant (tier 2) isolate SIVsmE660.CR54 as well as the primary isolate HIV-2_7312A_ (Fig 4). ITS02 was unique among the CD4bs-specific mAbs for its strain-specific neutralization of SIVmac251.H9 but not SIVsmE660, SIVmac251.30 or HIV-2_7312A_. None of the CD4bs-specific mAbs cross-neutralized the highly neutralization-resistant (tier 3) SIVmac239 (S1 Fig). As previously reported by other groups we observed that neutralization curves of tier 2 isolates of SIVsmE660 and SIVmac251 plateaued below 100%, and in some instances, below 50% neutralization despite using clonal, pseudo-typed viruses [39, 56, 82]. In order to compare the potency of individual mAbs, irrespective of neutralization plateau levels, we also calculated half-maximal (HalfMax) concentrations (i.e., the concentration required to achieve half-maximal neutralization) as well as maximum percent neutralization (VMax) values (i.e. the maximum % neutralization over the range of mAb concentrations tested) for individual mAbs (Fig 4). Based on these values, we determined that ITS01 and ITS20 were also weakly neutralizing against tier 2 SIVmac251.30 and that the potency of individual CD4bs mAbs was similar irrespective of the VMax levels.

### Antibodies targeting the cyanovirin binding site of SIV

**Fig 5:**
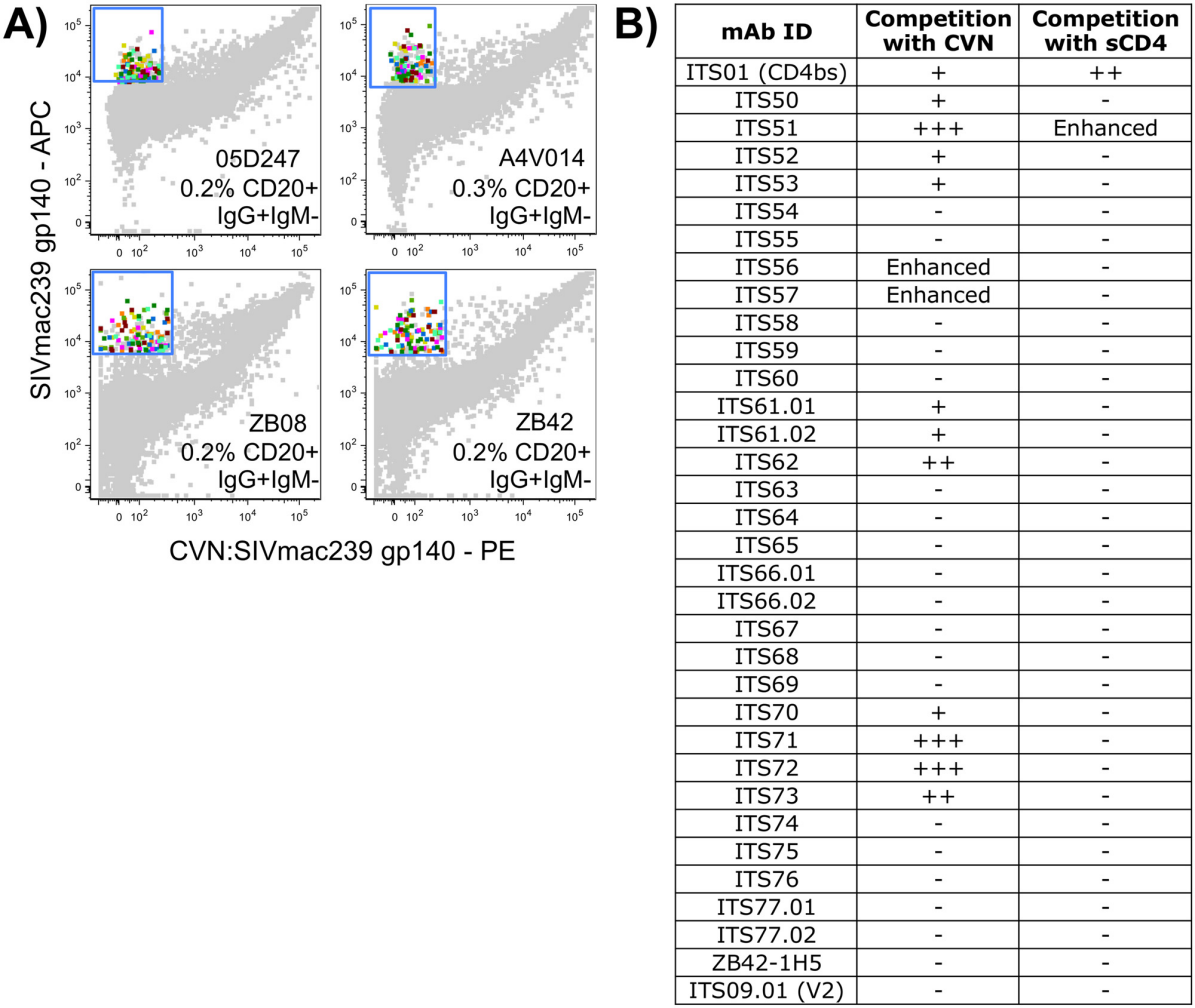
Isolation of CVNbs-specific B cells. A) FACS data overlay of individually sorted CVNbs-specific B cells (multicolored) from 4 SIV-infected rhesus macaques as a percentage of total memory B cells (gray). B) Summary of competition ELISA of individual mAbs showing percent inhibition: 75–100% competition (+++); 50–74% competition (++); 25–49% competition (+); <25% competition (-) or increased binding (Enhanced) in the presence of competing CVN.

Given the efficiency of our CD4bs competitive binding sort technique, we used the same strategy to isolate antibodies specific for the SIV Env glycan targets of cyanovirin (CVN), a potent inhibitor of primary and lab-adapted isolates of HIV and SIV [83]. Cyanovirin selectively binds to Man_8_ D1D3 and Man_9_ residues on N-linked glycans present on gp120 [67]. Binding of CVN also occludes the unique 2G12 neutralization epitope of HIV-1 Env gp120 [84]. We sorted putative cyanovirin binding site (CVNbs)-specific B cells from 4 SIVsmE660-infected rhesus macaques (05D247, A4V014, ZB08, ZB42) [35] (S1 Table; Fig 5A) and cloned and expressed a total of 32 mAbs from these 4 macaques (Table 2). Although 29 out of 32 mAbs cloned from the CVNbs competition sort bound to SIVmac239 gp140 FT (Fig 3), only 11 of these mAbs competed with CVN for binding to gp140 (Fig 5B). Intriguingly, the presence of CVN rescued the binding of 2 mAbs (ITS56 and ITS57) that failed to bind to SIVmac239 gp140 FT alone suggesting recognition of an epitope on both gp140 and CVN that is only present when they are bound together or conformational change(s) induced upon CVN binding that facilitated binding by these mAbs.

**Table 2:** Genetic characteristics of mAbs isolated from CVNbs competition B cell sort.

mAb ID	Animal	Rhesus heavy chain V gene	% Divergence (nt) from germline	CDRH3	Rhesus light chain V gene	% Divergence (nt) from germline	CDRL3
ITS50	05D247	IGHV4L*03	1%	ARANYEDDYGYYYKWGGVFDY	IGKV1-21*01	1%	QQYDDLPYS
ITS51	05D247	IGHV1E*01	4%	AASRSGNWYFDL	IGLV4S4*01	3%	QTWTNGIVL
ITS52	05D247	IGHV4A*02	5%	ACPLSGGLNYVLDV	IGKV1-21*01	8%	LQYTTSPWT
ITS53	05D247	IGHV3W*02	5%	ATMDL	IGKV2S18*01	9%	MQTLQTPFS
ITS54	05D247	IGHV4I*01	4%	ARLTVRRLDV	IGKV1S24*01	10%	QHGYGTPLT
ITS55	05D247	IGHV4L*03	6%	AREIGTTIIFRE	IGKV1S26*01	4%	QQGNSKPFT
ITS56	A4V014	IGHV1I*01	0%	ARAQGVYYEDDYGLYFDY	IGLV5-7*01	1%	TWHGNSKTVL
ITS57	A4V014	IGHV3K*01	7%	AMGSGGHSSRDVFDF	IGKV1-13*01	2%	LQGYDPPYS
ITS58	A4V014	IGHV7B*01	1%	ARWDWILQSLDWRVNSLDV	IGKV2S5*01	8%	MQGTHFPLT
ITS59	A4V014	IGHV3W*01	4%	TRDLPYTSWCRGDY	IGLV1-15*01	7%	AAWDDSLSGVL
ITS60	A4V014	IGHV4F*01	1%	ARAFWGYEDDYGYSDNGVYFDS	IGLV3-1*01	4%	QVWDFSSDHPI
ITS61.01	ZB08	IGHV3K*01	7%	VKGMRGDHEVESFEQIIAADPQGDV	IGKV2S17*01	5%	MQGVEFPWT
ITS61.02	ZB08	IGHV3K*01	8%	VKGMRGDIEVESFAQIIAADPAGDV	IGKV2S17*01	4%	MQGVEFPWT
ITS62	ZB08	IGHV4F*01	11%	ASILTGLEFDF	IGLV3-1*01	10%	QVWDISGDHMF
ITS63	ZB08	IGHV4F*01	8%	ASGYSGYSPFDY	IGKV1S9*01	12%	QHNYGTPWT
ITS64	ZB08	IGHV4F*01	7%	ARDSGIAAGEFDY	IGKV1-22*01	8%	QQYNSSPFT
ITS65	ZB08	IGHV3D*01	2%	AGDYDYGSNFLVDY	IGKV2S11*01	2%	VQVIAFPFT
ITS66.01	ZB08	IGHV3J*01	3%	ATGGWLLPFGY	IGLV1S1*01	8%	GAWDSSLSAGL
ITS66.02	ZB08	IGHV3J*01	5%	STGGWLLPFGY	IGLV1-13*01	6%	GAWDSSLSAGL
ITS67	ZB08	IGHV4F*01	7%	ARDGEATFDS	IGKV3-8*01	1%	QQENSTPT
ITS68	ZB08	IGHV7B*01	2%	ARQSRENTGFDY	IGKV3S6*01	1%	QQESNWSLT
ITS69	ZB08	IGHV1F*02	1%	ARTGIQWAQLDLGENYY	IGLV3-6*01	5%	QVWDSSSKYVL
ITS70	ZB42	IGHV4L*03	6%	ARDLVDSEYEVVWFDV	IGLV1-13*01	3%	GAWDTSLSARV
ITS71	ZB42	IGHV3L*01	5%	TRGSGWSEGNEEYFEF	IGLV6-5*01	6%	QSFDSNTYWL
ITS72	ZB42	IGHV1E*02	5%	ARGPRYEDDYGYYDWYFDL	IGLV3-1*01	7%	QVWDLSSDHVL
ITS73	ZB42	IGHV3L*01	3%	TRGNNFWSGSSHYFDY	IGLV1-7*01	5%	QSYDSSLSVHWV
ITS74	ZB42	IGHV3K*01	2%	AKDLPEYCSGSGCYAAPFDY	IGKV2-3*01	3%	MQALQAPYS
ITS75	ZB42	IGHV4E*02	2%	ARHLGGLNYGGRFDF	IGKV1-22*01	7%	LQYSSSPFT
ITS76	ZB42	IGHV3A*01	4%	ARDRSIAAATYYFDY	IGLV2S6*01	4%	NSYAGSNTFI
ITS77.01	ZB42	IGHV4L*03	6%	ARVPGIWFSKYYTFDF	IGLV2S9*01	6%	GSYREGSTFI
ITS77.02	ZB42	IGHV4L*03	7%	ARVPGIWFTNYYVFDF	IGLV2S9*01	7%	GSYRDGSTFI
ZB42.1H5	ZB42	IGHV4A*02	5%	ATIVVAVSAISWFDV	IGLV1-10*01	2%	SAWDSSLSGVL

### Antibodies isolated by CVNbs competition primarily target nonlinear Env epitopes

**Fig 6:**
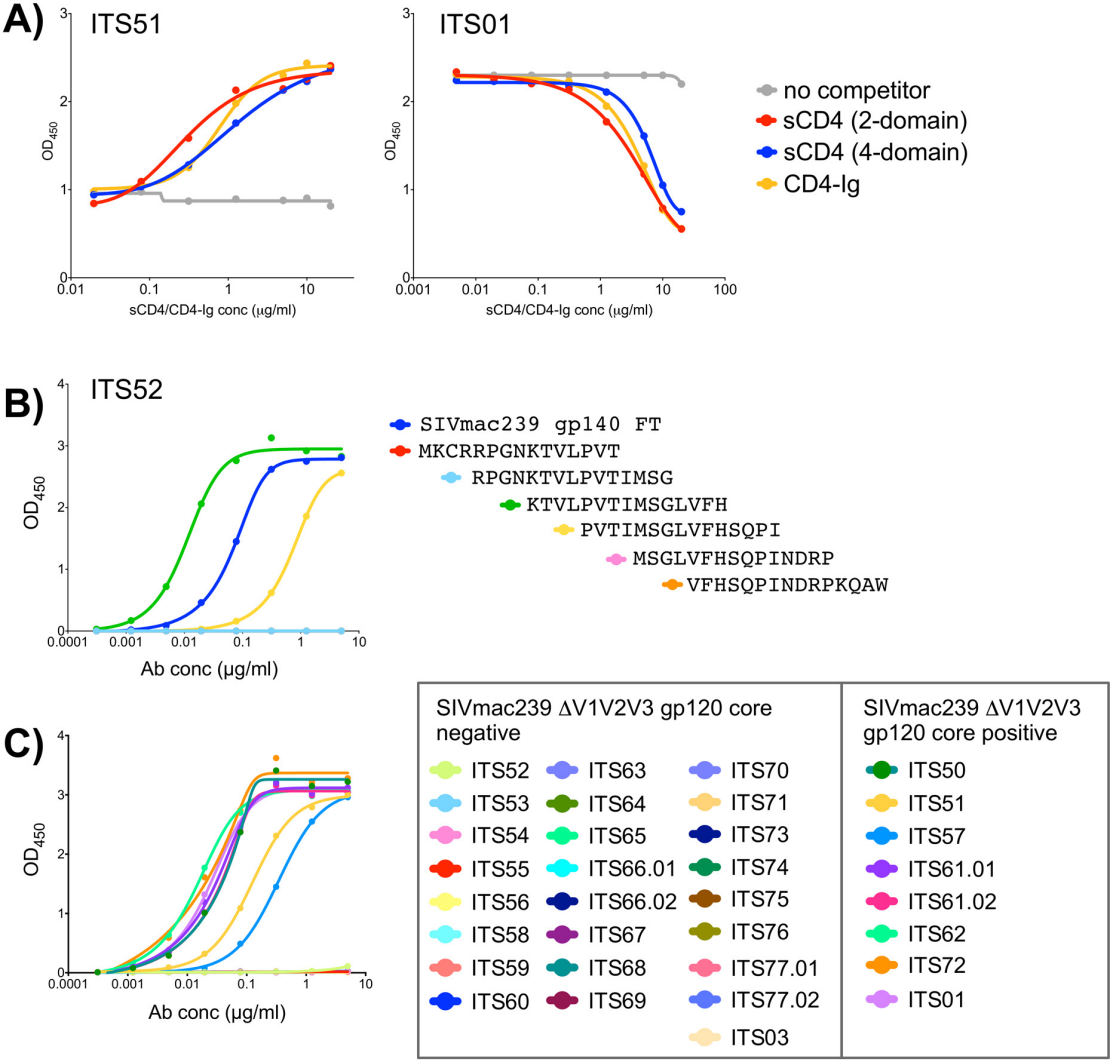
Mapping of CVNbs-specific mAbs. A) Competition ELISA of ITS51 and ITS01 (CD4bs) binding to SIVmac239 gp140 FT in the presence or absence of competing sCD4 or CD4-Ig. B) ELISA binding of ITS52 to SIVmac239 Env 15-mer overlapping peptides. C) ELISA binding curves showing SIVmac239 ΔV1V2V3 gp120 core-binding data of mAbs isolated from CVNbs competition B cell sort and control SIV mAbs.

The anti-HIV-1 activity of CVN is reportedly mediated through high affinity interactions with oligomannose residues suggesting multiple potential binding sites [67]. However, previous studies have reported that binding of CVN occludes subsequent binding of the bnAb 2G12 but not mAbs targeting the V3 and V4 loops, C4 region, CD4bs or CD4i epitopes of HIV-1 indicating a more defined binding epitope for CVN [84]. To map the SIV epitope(s) targeted by mAbs isolated by CVNbs competition we also assessed competition between these mAbs and sCD4. The presence of competing sCD4 did not block binding of any mAbs isolated by CVNbs competition (Fig 5B). However, one mAb (ITS51) showed enhanced binding to SIVmac239 gp140 FT in the presence of sCD4 (Fig 6A) indicating this mAb likely targets a CD4i site. Additionally, we tested mAbs from this sort for binding to overlapping SIVmac239 Env 15-mer peptides. Of 31 mAbs that exhibited SIV gp140 FT binding, 30 were negative for binding to SIVmac239 Env peptides. Only ITS52 was mapped to a linear peptide sequence near the V3 loop tip (Fig 6B). To further evaluate the epitope binding specificities of mAbs isolated by CVNbs competition, we tested binding of individual mAbs to a SIVmac239 ΔV1V2V3 gp120 core protein generated by deletion of the V1V2 loops and truncation of the V3 loop [63]. Of 31 mAbs that bound to SIVmac239 gp140 FT, 7 were also positive for binding to SIVmac239 ΔV1V2V3 gp120 core protein (Fig 6C). As expected, ITS01, a CD4bs-specific mAb also bound to ΔV1V2V3 gp120 core protein while V2-specific (ITS03) and V3-specific (ITS52) mAbs did not.

**Fig 7:**
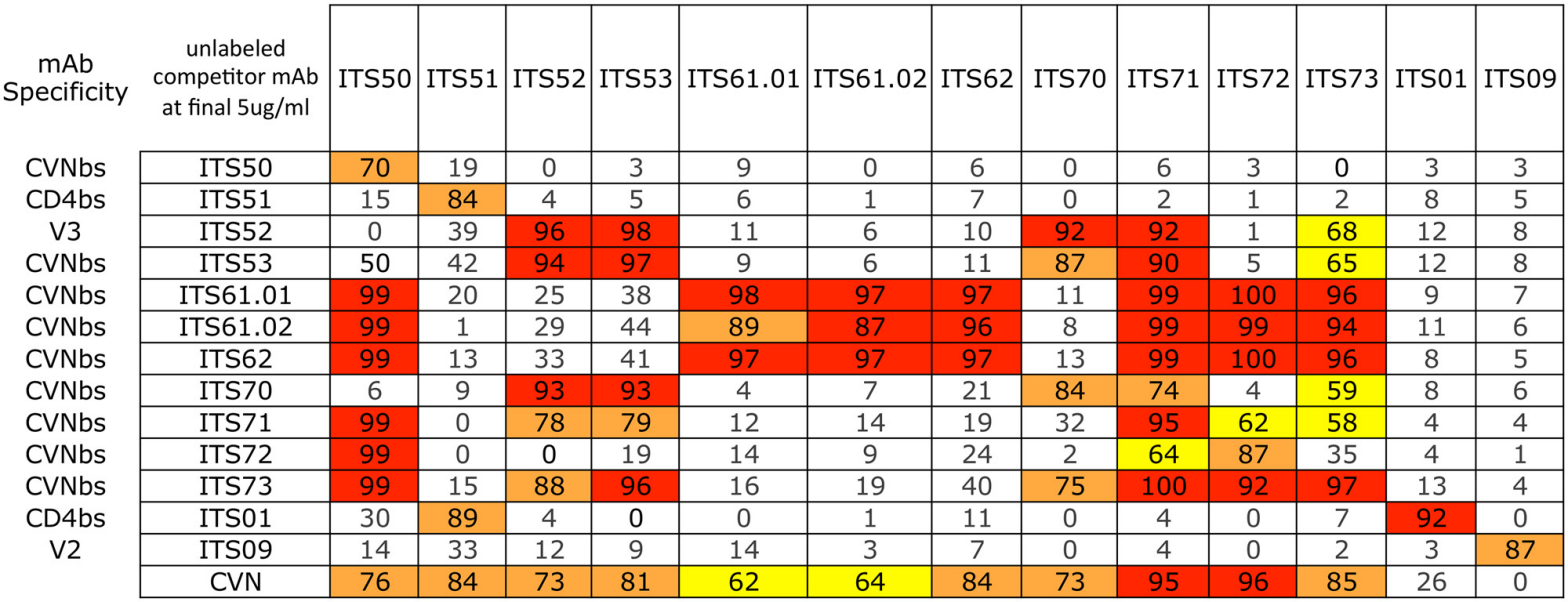
Cross-competition patterns between selected CVNbs mAbs. The effects of unlabeled competitor mAb/protein (listed in the 2^nd^ column) on the binding of biotin-labeled mAbs (listed across the top) to plate-bound SIVmac239 gp140 FT were measured in duplicate. Values indicate percent inhibition (average OD reading in the absence of competitor ligand minus average OD reading in the presence of competitor ligand) / average OD value in control wells expressed as a percentage). Values with insignificant competition are unshaded. Values with low (50–69%), intermediate (70–89%) and high (≥90%) levels of competition are indicated as yellow, orange and red boxes, respectively.

To assess overlapping SIV Env epitope-binding specificities of ITS52 and other mAbs isolated by CVNbs competition, we performed a matrix cross-competition ELISA of CVNbs mAbs. For 11 CVNbs mAbs tested there were multiple patterns of cross-competition although most were competed efficiently by multiple mAbs suggesting overlapping specificities (Fig 7). The ability of ITS01 to compete with ITS51 for binding to SIVmac239 gp140 FT, together with the sCD4 and CD4-Ig competition data (Fig 6A), indicate that ITS51 targets the CD4i-site of SIV. Overall, our CVNbs competition sort yielded mAbs targeting the V3 loop, CD4i-site and ΔV1V2V3 SIVmac239 gp120 core protein.

### Antibodies isolated by CVNbs competition neutralize SIV with high potency

Cyanovirin has been reported to neutralize HIV-1, HIV-2 and SIV primary isolates at low nanomolar concentrations [67, 83]. To determine whether mAbs isolated by CVNbs competition could mediate similar virus neutralization breadth and potency, we assessed neutralization activity of all 32 mAbs isolated by the CVNbs competition sort against a small panel of SIV Env pseudoviruses and HIV-2_7312A_. Nineteen mAbs were non-neutralizing against all 5 viruses tested while 13 mAbs neutralized SIVsmE660.CP3C (tier 1), SIVsmE660.CR54 (tier 2) and SIVmac251.H9 (tier 1) (Fig 4). Of these, 8 mAbs also neutralized SIVmac251.30 (tier 2) and 3 cross-neutralized HIV-2_7312A_. Thus, CVNbs mAbs showed similar virus neutralization cross-reactivity as CD4bs mAbs; however, the neutralization potency of several CVNbs mAbs was significantly higher than that of CD4bs mAbs. In some instances 10,000-fold lower IC_50_ (1,000-fold lower HalfMax) values were obtained for CVNbs mAbs compared with the most potent neutralizing CD4bs mAbs (Fig 4). Of note, neutralizing activity correlated with specificity for the CVNbs. All 11 mAbs which efficiently competed with CVN for binding to SIVmac239 gp140 FT were neutralizing while of the remaining non-CVNbs-specific mAbs tested, only ITS56 and ITS57, which required the presence of CVN for binding to SIVmac239 gp140 FT, were neutralizing (Figs 4 and 5B).

### Isolation of V1V2-specific antibodies

**Fig 8:**
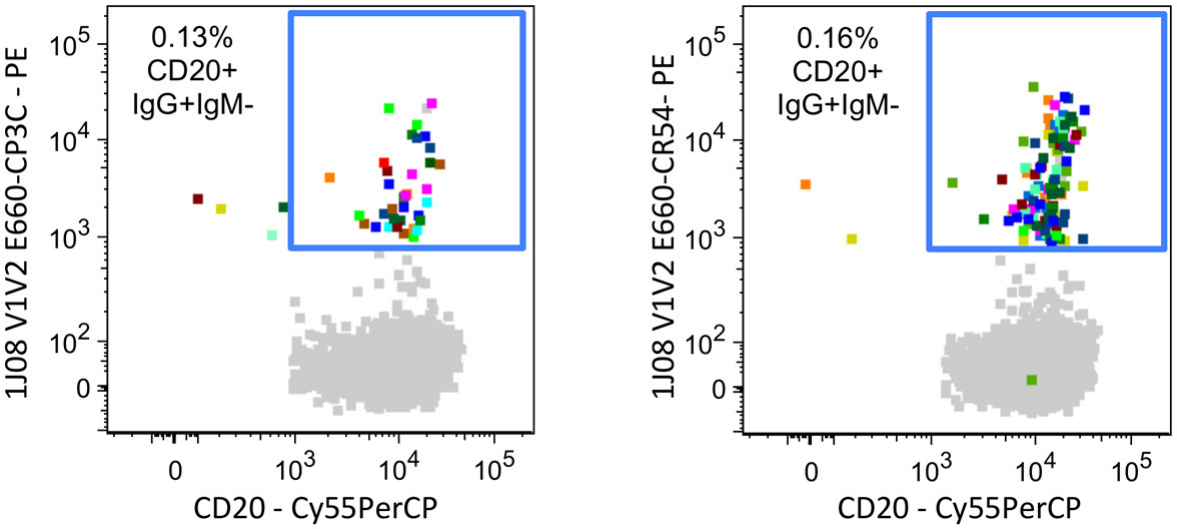
Isolation SIV V1V2-specific B cells by FACS. FACS data overlays showing individually sorted (multicolored) 1JO8 SIVsmE660.CP3C (left) and 1JO8 SIVsmE660.CR54 (right) V1V2-specific B cells from a SIV-infected rhesus macaque as a percentage of total memory B cells (gray).

Both human and non-human primate studies have shown that V1V2 serum IgG binding levels correlate with protection against HIV/SIV infection [36–38, 85]; therefore, we were also interested in isolating SIV V1V2-specific B cells. We generated 1JO8-scaffolded SIV V1V2 probes [60] from tier 1 (SIVsmE660.CP3C) and tier 2 (SIVsmE660.CR54) isolates of the SIVsmE660 challenge swarm used in a recently completed SIV challenge study [38]. Individual fluorescently labeled 1JO8-scaffolded SIV V1V2 probes were used to stain and isolate V1V2-specific B cells from a SIVmac239-vaccinated and SIVsmE660-infected rhesus macaque (S1 Table). We sorted 36 (0.13% of memory B cells) and 110 (0.16% of memory B cells) SIV V1V2-specific B cells using 1JO8-scaffolded SIVsmE660.CP3C and SIVsmE660.CR54 V1V2 probes, respectively (Fig 8). A total of 26 mAbs belonging to 9 distinct clonal families were cloned from 146 individually sorted cells and some of these represented identical clones (Table 3). There were 20 unique mAbs: 3 identical clones of ITS03 and 2 identical clones for each of ITS09.01, ITS10.04, ITS12.01 and ITS30.

**Table 3:** Genetic characteristics of mAbs isolated from animal 08D038, a SIVmac239-vaccinated, SIVsmE660-challenged rhesus macaque using 1JO8 SIVsmE660 V1V2-scaffolded probes.

mAb ID	1JO8 SIV V1V2 sort probe	Rhesus heavy V chain gene	% Divergence (nt) from germline	CDRH3	Rhesus light chain V gene	% Divergence (nt) from germline	CDRL3
ITS03	E660.CR54	IGHV1J*01	5%	ATEGTAAPTAF	IGKV4-3*01	2%	QQYYSTRLT
ITS06.01	E660.CR54	IGHV3Q*02	8%	ARWASRGYFDY	IGKV2S17*01	6%	MQVLDFPFT
ITS06.02	E660.CP3C	IGHV3Q*02	9%	ARWASRGYFDS	IGKV2S17*01	6%	MQVLDFPFT
ITS09.01	E660.CP3C	IGHV1J*01	9%	ATDPEYNCA	IGKV4-3*01	7%	QQYLSSPFT
ITS09.02	E660.CR54	IGHV1J*01	5%	ATDPEYGCT	IGKV4-3*01	3%	QQYYRSPFT
ITS09.03	E660.CR54	IGHV1J*01	11%	ATDPEYGCT	IGKV4-3*01	9%	QQYLNAPFT
ITS09.04	E660.CR54	IGHV1J*01	10%	ATDPGYGCT	IGKV4-3*01	7%	QQYLNAPFT
ITS10.01	E660.CP3C	IGHV3AB*01	9%	TRGSGYNVY	IGKV3S3*01	12%	QQFAKWPHG
ITS10.02	E660.CR54	IGHV3AB*01	8%	TRGSGYNLY	IGKV3S3*01	10%	QQFANWPHG
ITS10.03	E660.CR54	IGHV3AB*01	9%	TRGSGYNVY	IGKV3S3*01	12%	QQFAKWPHG
ITS10.04	E660.CR54	IGHV3AB*01	9%	TRGSGYNIY	IGKV3S3*01	10%	QQFAKWPHG
ITS12.01	E660.CP3C	IGHV3AB*01	9%	ISQEVSGSYHYFDY	IGKV2S18*01	4%	MQALRSPWT
ITS12.02	E660.CR54	IGHV3AB*01	9%	ISQEVSGSYHYFDY	IGKV2S18*01	4%	MQALRSPWT
ITS13	E660.CR54	IGHV3Q*02	13%	ARVGVAADKRYSFIDS	IGKV2S13*01	14%	MQSKEFPFT
ITS30	E660.CP3C	IGHV3AB*01	13%	TRGSGYNIY	IGKV3S3*01	9%	QQFSSWPHDV
ITS34.01	E660.CP3C	IGHV3AB*01	7%	ARGSGCSIY	IGKV3-4*01	8%	QQYSNWPHG
ITS34.02	E660.CR54	IGHV3AB*01	8%	ARGSGCSIY	IGKV3-4*01	6%	QQYSNWPHG
ITS34.03	E660.CR54	IGHV3AB*01	8%	ARGSGCSIY	IGKV3-4*01	10%	QQYSDWPHG
ITS37	E660.CR54	IGHV3AB*01		VRGSGCNLF	IGKV3-4*01	10%	QQYNDWPHG
08D038.2C2	E660.CR54	IGHV3J*02	8%	TRPYYSGSYYWDY	IGKV3-2*01	4%	QKYSNSPYS

### Binding and neutralization targets of SIV V1V2-specific mAbs

**Fig 9:**
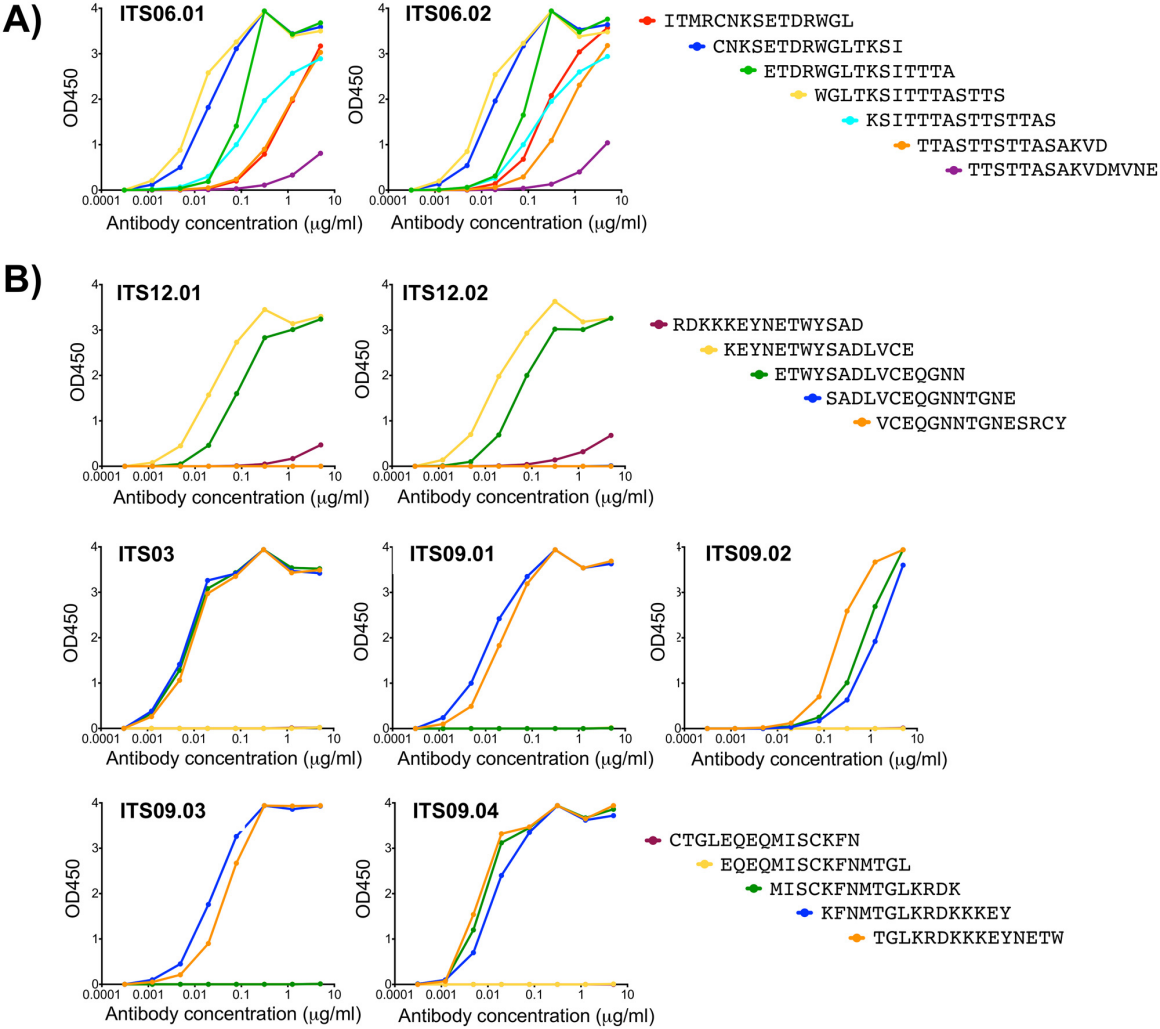
Epitope mapping of SIV-specific mAbs. ELISA testing of SIV V1V2-specific mAbs binding to SIVmac239 Env 15-mer overlapping peptides within A) V1 and B) V2 regions.

The 1JO8 SIVsmE660 probes were highly specific since only one of 20 mAbs expressed did not bind to SIVsmE660 gp120 (Fig 3). The remaining 19 mAbs bound to both SIVsmE660.CP3C and SIVsmE660.CR54 gp120s with several also binding to SIVmac251.30 gp120 and SIVmac239 gp140 FT. The epitope binding specificities of SIV V1V2 mAbs were assessed by ELISA binding using overlapping 15-mer SIVmac239 Env peptides spanning the V1V2 region. Peptide mapping revealed that ITS06.01 and ITS06.02 bound to several peptides from V1 corresponding to Env 109–147 (SIVmac239 numbering) (Fig 9A) suggesting a potentially discontinuous epitope within this region while ITS12.01 and ITS12.02 targeted Env 185–195 (ETWYSADLVCE) (Fig 9B), an epitope at the C-terminus of the V2 loop. Interestingly, although both of these mAbs bound to this SIVmac239 peptide epitope, neither mAb bound to either SIVmac239 gp140 FT or SIVmac251.30 gp120. Another linear B cell epitope in V2 was identified by ITS03 and ITS09.01–04 mAbs which bound within Env 173–183 (TGLKRDKKKEY) (Fig 9B). While Env 173–179 was sufficient for binding by ITS03, ITS09.02 and ITS09.04, additional residues were necessary for binding by ITS09.01 and ITS09.03. All mAbs which did not bind to either SIVmac239 gp140 FT or 15-mer peptides were screened for binding to potentially protective SIVsmE660 15-mer peptide sequences [38]. These mAbs all bound to a 15-mer peptide corresponding to Env 142–156 (ENVINESNPCIKNNS), an epitope that is present in SIVsmE660 but not SIVmac239 (S2 Fig).

We also assessed neutralizing activity of SIV V1V2-specific mAbs against tier 1 and 2 clonal isolates of SIVsmE660 and SIVmac251 and HIV-2_7312A_. Despite strong binding to peptide ENVINESNPCIKNNS, which is present in SIVsmE660.CP3C and SIVsmE660.CR54, only 4 of 9 mAbs specific for this epitope showed weak neutralization against SIVsmE660.CP3C (Fig 4). Likewise, ETWYSADLVCE-specific mAbs ITS12.01 and ITS12.02 showed strain-specific neutralization of SIVsmE660 isolates but not SIVmac251 or SIVmac239 despite strong binding to SIVmac239 gp140 FT and linear peptides. In contrast, ETDRWGLTKSI-specific mAbs ITS06.01, ITS06.02 and ITS13 were cross-neutralizing for SIVsmE660 and SIVmac251 isolates albeit with varying degrees of breadth and potency. ITS06.02 and ITS13 neutralized tier 1 and tier 2 isolates of SIVsmE660 and the tier 1 isolate SIVmac251.H9 but not SIVmac251.30 (tier 2). ITS06.01 was the only V1-specific mAb which neutralized both tier 1 and 2 isolates of SIVsmE660 and SIVmac251. Three V2-specific mAbs targeting the TGLKRDKKKEY epitope (ITS03, ITS09.03 and ITS09.04) were also neutralizing against the same 4 isolates. None of the V1V2-specific mAbs neutralized SIVmac239 or HIV-2_7312A_.

### 1JO8-scaffolded SIV V1V2 probes identify heterologous vaccine-elicited B cells

**Fig 10:**
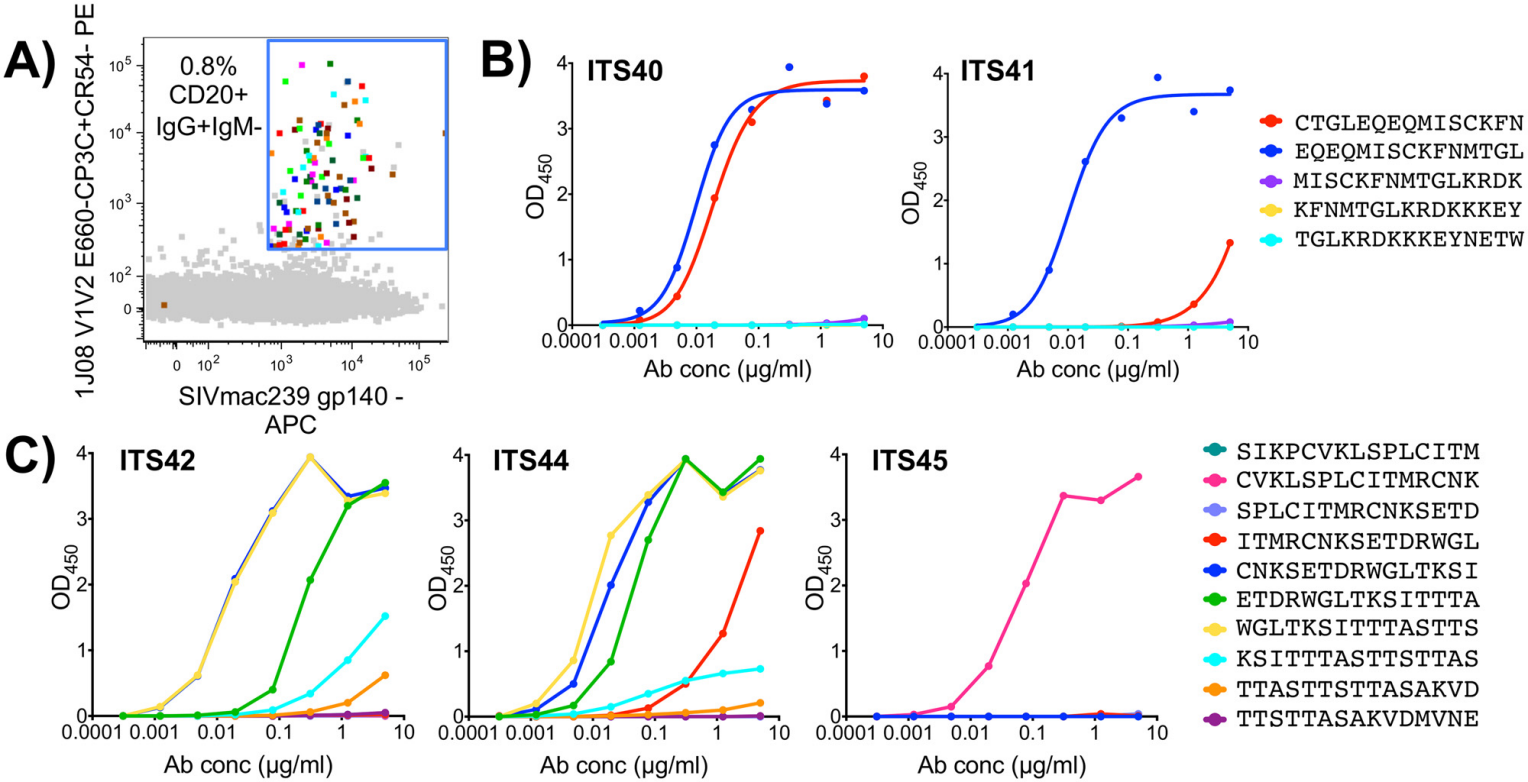
Vaccine-elicited SIV V1V2-specific B cells and mAbs. A) FACS data overlay showing vaccine-elicited (multicolored) 1JO8 SIVsmE660.CP3C and SIVsmE660.CR54 V1V2-specific B cells from a pre-challenge, SIV vaccinated rhesus macaque as a percentage of total memory B cells (gray). ELISA testing of vaccine-elicited SIV V1V2-specific B mAbs binding to SIVmac239 Env 15-mer overlapping peptides from B) V2 or C) V1.

The 1JO8-scaffolded V1V2 probes efficiently labeled V1V2-specific B cells from the chronic phase of SIV infection; however, we wanted to determine whether these probes could also be used to isolate low-frequency vaccine-elicited B cells that might be cross-reactive for heterologous challenge virus. We used the 1JO8 SIVsmE660 V1V2 probes to sort B cells from a pre-challenge, SIVmac239-vaccinated macaque (ZG12) [38] (S1 Table). In order to maximize probe binding to heterologous, low frequency pre-challenge memory B cells, we used both 1JO8 SIVsmE660.CP3C and SIVsmE660.CR54 V1V2 probes in combination to sort 74 (0.8% of memory B cells) SIV V1V2-specific B cells (Fig 10A). We cloned 7 unique mAbs belonging to 6 clonal families (Table 4) and characterized their binding specificities and neutralization activity. Six out of 7 mAbs tested bound to the 1JO8 SIV probes used for cell sorting and to SIVmac239 gp140 FT (Fig 3). Peptide mapping revealed that ZG12-2H10, which failed to bind SIVmac239 gp140 FT, did not bind to any SIVmac239 Env 15-mer overlapping peptides and was non-neutralizing against all viruses tested (Fig 4). Five mAbs were mapped to 1 of 3 epitopes including two new epitopes not identified by infection-related V1V2 mAbs (Fig 3). Both ITS40 and ITS41 which targeted the V2 epitope EQEQMISCKFNMTGL (Fig 10B), only neutralized tier 1 SIVsmE660.CP3C (Fig 4) while ITS45 targeted Env 101–115 (CVKLSPLCITMRCNK) (Fig 10C) but was non-neutralizing against all isolates tested. Similar to ITS06.01 and ITS06.02 mAbs isolated from chronic SIV infection, ITS42 and ITS44, bound to several peptides from V1 corresponding to Env 109–147 (Fig 10C) and neutralized tier 1 isolates of SIVsmE660 and SIVmac251 as well as the tier 2 isolate SIVsmE660.CR54 (Fig 4).

**Table 4:** Genetic characteristics of mAbs isolated from animal ZG12, a SIV-vaccinated, unchallenged rhesus macaque using 1JO8 SIV V1V2-scaffolded probe.

mAb ID	Rhesus heavy chain V gene	% Divergence (nt) from germline	CDRH3	Rhesus light chain V gene	% Divergence (nt) from germline	CDRL3
ITS40	IGHV4L*03	10%	VRLSLVGLDS	IGLV1S6*01	3%	GTWDSSLSAWV
ITS41	IGHV4L*03	11%	ARLGLIGVDY	IGLV1S6*01	4%	GTWDTSLSAWV
ITS42	IGHV2A*01	5%	ARSALTGVTSILDS	IGLV3S16*01	12%	QSADISDNLL
ITS43	IGHV3Q*02	4%	ARWGCTGSGCYASFDY	IGKV2S17*01	2%	MQGLEFPPR
ITS44	IGHV3Q*02	13%	VRELFYGGSYFFYN	IGLV3S15*01	14%	QSLDITGSYPF
ITS45	IGHV3L*01	3%	SRGENFWSGYSTEYWFDV	IGLV3S16*01	11%	QSADFSGNHWI
ZG12.2H10	IGHV3AB*01	8%	TRVLYSEDFDY	IGKV2S4*01	4%	GQGAHWPWT

### Neutralization breadth and potency of SIV mAbs

**Fig 11:**
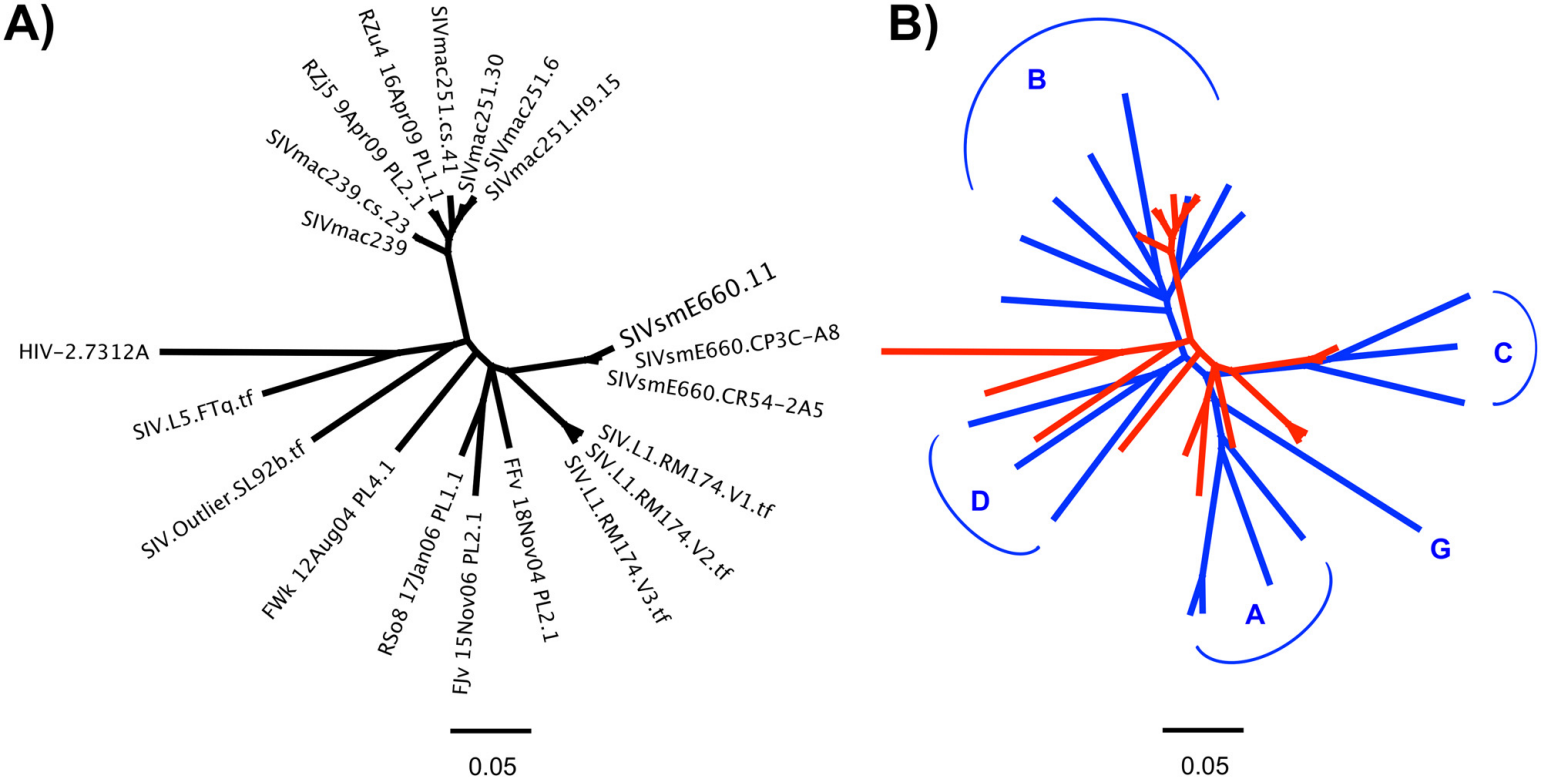
Genetic diversity of SIV and HIV Envs. Phylogenetic tree depicting (A) genetic diversity of SIV Env sequences from a 21 virus panel used to assess SIV mAb neutralization breadth and potency. (B) Overlay of phylogenetic trees depicting genetic diversity of Env sequences from a multi-clade (indicated in blue letters) panel of 19 HIV-1 pseudoviruses (blue) and a panel of 20 SIV viral isolates as well as HIV-2 isolate 7312A (red). Tree topology was inferred from the neighbor-joining method based on ClustalW alignment of protein sequences.

**Fig 12:**
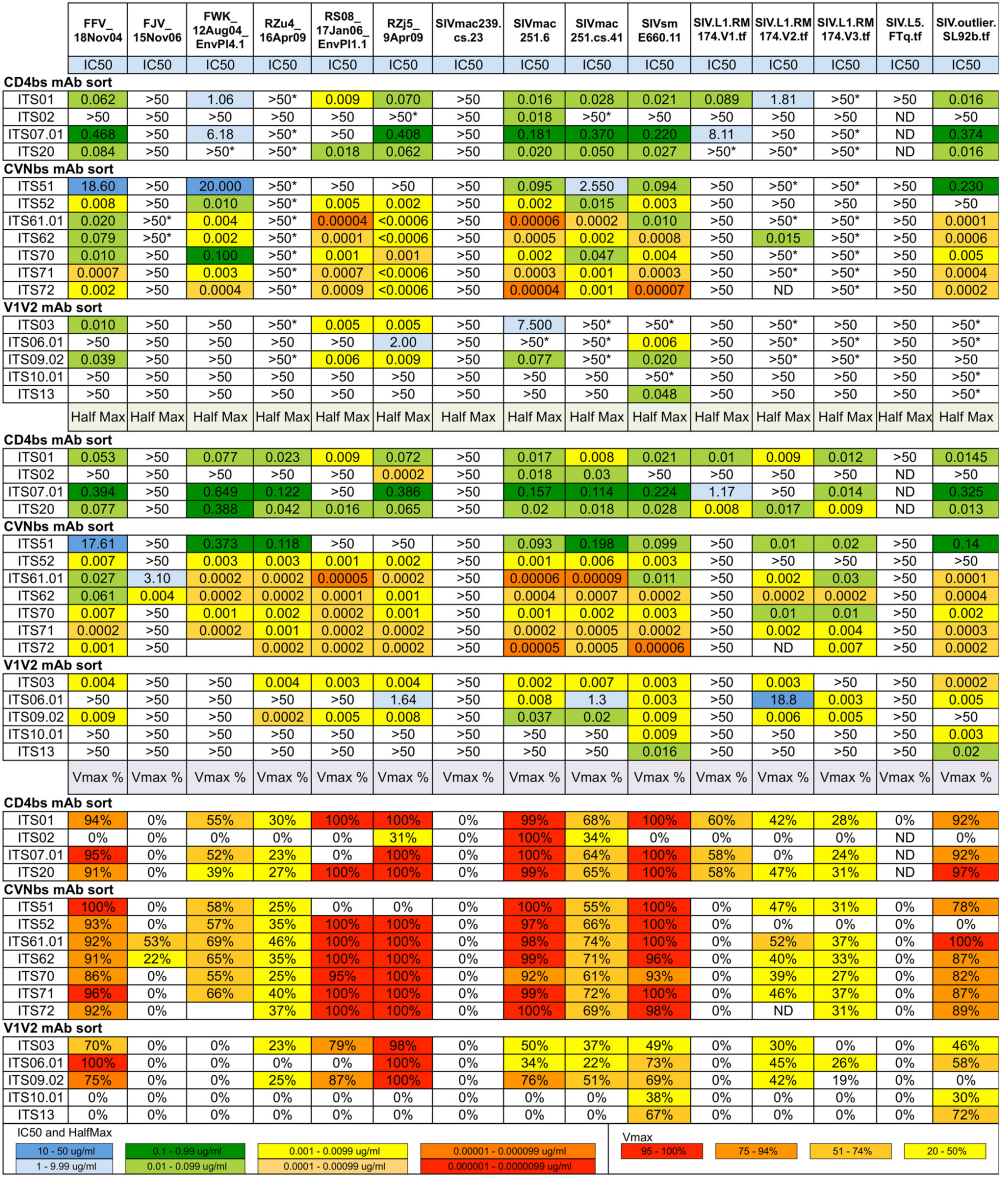
Breadth of SIV-specific mAbs. mAbs cloned from individually sorted SIV Env-specific B cells were evaluated for neutralization of SIV pseudoviruses and transmitted founder (TF) virus IMCs. Values >50 indicate no neutralization while >50* indicates neutralization curve plateaued below 50%. ND = not determined.

**Fig 13:**
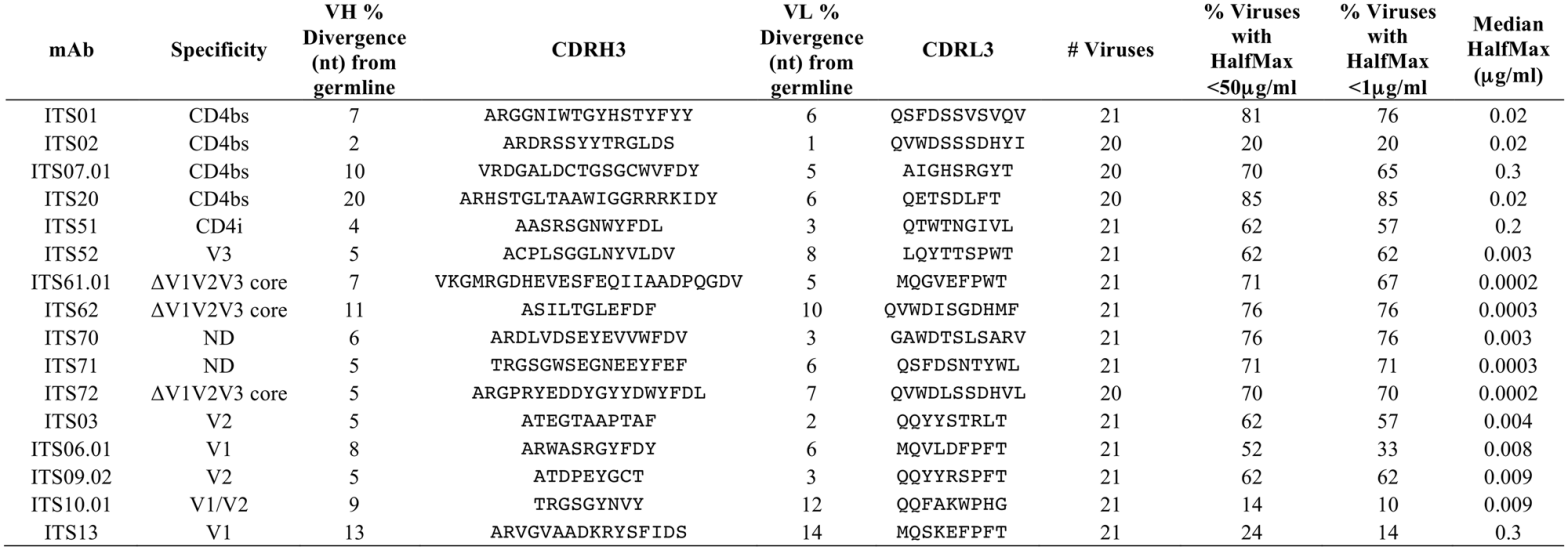
Neutralizing activity of SIV mAbs against a 21-virus panel. The percentage of viruses neutralized with half maximal neutralization (HalfMax) <50 μg/ml and <1 μg/ml as well as the median HalfMax values for viruses neutralized with HalfMax < 50 μg/ml.

While several SIV mAbs were cross-neutralizing for SIVmac251 and SIVsmE660 isolates tested, the small panel of closely related SIV Env pseudoviruses used to test neutralization activity limits our ability to assess neutralization breadth of individual SIV mAbs. Therefore, we tested selected SIV mAbs for neutralization against an additional 15 SIV isolates including 10 SIV Env pseudoviruses and 5 transmitted/founder IMCs (Fig 11). Combined with the SIVmac251, SIVsmE660 and HIV-2_7312A_ viral isolates initially tested (Fig 4), this expanded 21-virus panel (Fig 11A) more closely reflects inter-clade genetic diversity of HIV-1 (Fig 11B) [80]. Most SIV mAbs tested showed neutralization of multiple SIV strains including neutralization of genetically diverse tier 2 and tier 3 SIV isolates. (Fig 12). In general, SIV CD4bs and CVNbs mAbs displayed greater neutralization breadth than SIV V1V2 mAbs while CVNbs mAbs were among the most potent (Fig 13, S6–S8 Figs). Neutralization breadth, as measured by the percentage of SIV/HIV-2 isolates neutralized, was greatest for CD4bs mAbs ITS01 and ITS20, which neutralized up to 81% and 85% of viruses tested, respectively (Fig 13).

Unsurprisingly, strain-specific mAbs ITS02 (CD4bs) and ITS10.01 (V1) had the lowest neutralization breadth, 20% and 14%, respectively, while the neutralization breadth of CVNbs mAbs ranged from 62–76%.

Broadly neutralizing mAbs isolated from HIV-1 infected individuals exhibit some unique features such as high diversity in the variable heavy chain region (VH) genes due to extensive somatic hypermutation (SHM) [86] and long, protruding CDRH3 sequences [87]. The level of SHM for HIV-1 bnAbs ranges from 11–32% divergence from putative VH germline nucleotide sequence [88] while HIV-specific antibodies with low or no neutralizing activity display approximately 9–12% VH sequence divergence from germline [89, 90]. Compared with HIV-1 bnAbs or even rhesus memory B cells, which are approximately 5% divergent from VH germline nucleotide sequence [91], the SIV-specific bnAbs isolated here have relatively low levels of SHM. With regard to CDRH3 length, HIV-1 CD4bs bnAbs have relatively short CDRH3 sequences while those targeting quaternary bnAb epitopes of the V1/V2 and V3 loops have long CDRH3s likely to facilitate penetration of the glycan shield and access to the V1/V2 and V3 loops [12, 87]. Based on the distribution of CDRH3 length in rhesus naïve B cells [91], we did not observe unusually long CDRH3 sequences among SIV V1V2-specific mAbs and this was not altogether surprising since the V1V2 mAbs isolated in this study all targeted linear peptide epitopes and displayed limited neutralization breadth against our 21 virus panel. By comparison, some of the SIV CD4bs and CVNbs bnAbs had longer CDRH3 sequences; however, there is no structural data as yet to support the requirement for long CDRH3s to access recessed epitopes by SIV bnAbs as is the case for HIV-1 quaternary-preferring bnAbs with long CDRH3s. In general, neutralizing SIV mAbs did not display some of the unique features frequently observed in HIV-1 bnAbs.

## Discussion

The SIV NHP model for HIV-1 is useful for studying vaccine mediated and immune correlates of protection but little is known about binding or neutralizing epitopes on SIV Env. Our goal was to isolate and characterize SIV Env-specific mAbs that might facilitate effective use of this NHP model for understanding the variables in development of a HIV vaccine or immunotherapy. We demonstrate the use of a novel competitive probe binding strategy for the targeted isolation of SIV Env-specific mAbs from rhesus macaques and present a detailed assessment of nearly 70 SIV mAbs targeting the CD4bs, CD4i-site, CVNbs and V1, V2 and V3 loops of SIV Env. We characterized individual SIV mAbs with regard to immunoglobulin genetics, epitope specificity, peptide and protein binding as well as virus neutralization breadth and potency.

Various studies have characterized neutralization epitopes of SIV using murine, guinea pig, rabbit and goat antisera [92, 93] as well as murine- [94–98] or rhesus-derived [99–102] SIV-specific mAbs. The range of epitopes described includes SIV mAbs targeting linear epitopes in variable loops 1–4 [38, 92, 93, 95–97, 100–102] and conformational epitopes involving the V3-V4 region [100, 101] as well as those overlapping or proximal to the CD4bs [95, 98] and co-receptor binding site [95]. However, SIV-specific mAbs isolated to date have been produced exclusively from hybridomas [94–98], EBV-transformed B cells [99–101] or phage display [102]. Ours is the first study to describe the targeted isolation of SIV epitope-specific B cells from rhesus macaques using direct and indirect binding to novel SIV probes.

Given the paucity of reagents for the targeted isolation of SIV-specific B cells we developed a simple competitive probe binding strategy to sort CD4bs-directed B cells from which we cloned multiple SIV CD4bs mAbs. Nearly 70% of mAbs isolated by this method were confirmed to target the CD4bs—significantly improved efficiency as compared to the isolation of HIV CD4bs mAbs using HIV-1 resurfaced stabilized core 3 (RSC3) protein [5]. By substituting CVN in place of CD4-Ig as competitor ligand, we were able to modify the target cell population to sort CVNbs-specific B cells. We subsequently cloned several mAbs from sorted B cells and confirmed their specificity for the CVNbs, thereby validating this competitive probe binding strategy as a powerful technique for the targeted isolation of SIV-specific B cells. Based on the simplicity and efficiency of our competitive probe binding sort strategy we propose that this method may be preferable to the use of engineered probes for targeted isolation of epitope-specific B cells—at least epitopes for which probe binding ligands are available. With this method, even antibodies could serve as competitive ligands for Env trimer probes to facilitate isolation of additional antibodies targeting a given epitope without the need for time-consuming probe development. Competitive ligands cross-reactive for divergent HIV/SIV Env probes could extend the applicability of this competitive sort strategy to diverse strains of HIV/SIV. This simple and effective competitive probe sort technique may also prove useful for the isolation of virus-specific B cells in general. The 1JO8-scaffolded SIV V1V2 probes we designed and tested were also remarkably efficient at labeling both high frequency infection-related and low frequency vaccine-elicited SIV V1V2-specific B cells. Several of the SIV V1V2 epitopes targeted by mAbs isolated with the 1JO8-scaffolded SIV V1V2 probes have previously been identified following isolation of B cells using other strategies [103] thereby validating the use of these probes for targeted isolation of V1V2-specific B cells. Overall, the ability to efficiently target SIV mAbs of defined specificities will increase the usefulness and relevance of the SIV model for studying the induction and maturation of virus-specific B-cells.

Many SIV-specific B cell epitopes previously reported have been identified using murine derived or HIV-2-specific mAbs exhibiting cross-reactivity with SIV [103]. Here, we provide the most extensive study of SIV Env-specific mAbs isolated from rhesus macaques including the first reported rhesus SIV CD4bs-specific mAbs. Of nearly 70 SIV Env-specific mAbs isolated the most broadly neutralizing SIV mAbs were CD4bs-specific, likely due to the conserved nature of the CD4bs for maintaining functional contact with its primary receptor CD4. Indeed, cross-reactivity of HIV-2 CD4bs mAbs for SIV has previously been reported [39]. Among the CD4bs mAbs, ITS02 was notable for its strain-specific neutralization of SIVmac251 (tier 1) only, suggesting that despite strong competition with CD4-Ig for binding to SIV Env, its epitope is likely proximal to rather than directly at the CD4bs.

As with the CD4bs mAbs, CVNbs mAbs also displayed considerable neutralization breadth against our 21-virus panel; however, only a fraction of CVNbs mAbs cross-neutralized HIV-2. Given the lack of information regarding epitope specificity of most of the CVNbs mAbs it is unclear whether the lack of cross-reactivity for HIV-2 is due to sequence, glycosylation or other conformational differences between SIV and HIV-2 or some combination thereof. The single CVNbs mAb for which we determined peptide-binding specificity within the V3 loop, neutralized 62% of isolates tested despite minimal sequence variation in this epitope among SIVs and HIV-2_7312A_. Of note, several CVNbs mAbs were significantly more potent than either the SIV CD4bs- or V1V2-directed mAbs. Among these were 2 clonally related CVNbs mAbs (ITS61.01 and ITS61.02) with extraordinarily high potency and unusually long (25 residues) heavy chain complementarity determining region 3 (CDRH3) loops similar to V2 and V3 glycan reactive mAbs that are among the most potent HIV-1 bnAbs [9, 12, 104–106]; however, these high potency mAbs do not target V2 or V3 glycans since they bind to V1,V2,V3-deleted SIV gp120 core protein.

Compared with the relatively minor sequence variation between the V3 loops of SIV/HIV-2, there is considerable sequence diversity between SIV and HIV-2 within the V1 and V2 loops and this was reflected by the fact that all SIV V1V2 mAbs isolated were non-neutralizing against HIV-2. Interestingly, all SIV V1V2 mAbs were mapped to linear peptide epitopes although it is unclear whether this was due primarily to the 1JO8-scaffolded probe used for isolating B cells and/or the immunization history or immune response of animals used for cell sorting. Among the major sites of HIV-1 Env vulnerability, the V1V2 loops are of particular interest for an HIV vaccine based on results of both human and NHP vaccine efficacy trials showing that levels of V1V2-specific serum binding, but not neutralizing activity, directly correlate with resistance to HIV/SIV infection [38, 85]. Among the SIV V1V2 mAbs isolated we identified both neutralizing as well as binding, non-neutralizing mAbs which may serve as useful reagents for delineating the role of V1V2-binding mAbs towards protection against infection. An important caveat is the presence of two conserved cysteine residues in the V2 region of most SIV and HIV-2 strains, which are absent in all HIV-1 strains [107]. These twin-cysteine residues may form a disulfide bond that contributes to Env trimer stabilization since twin-cysteine mutants exhibit decreased gp120 association with the Env trimer cell-cell fusion and virus infectivity. Future studies will need to address whether the conserved twin-cysteine motif may contribute to structural or functional differences between SIV/HIV-2 and HIV-1 and any potential impact on V1V2-directed mAb responses in SIV/HIV-2.

**Fig 14:**
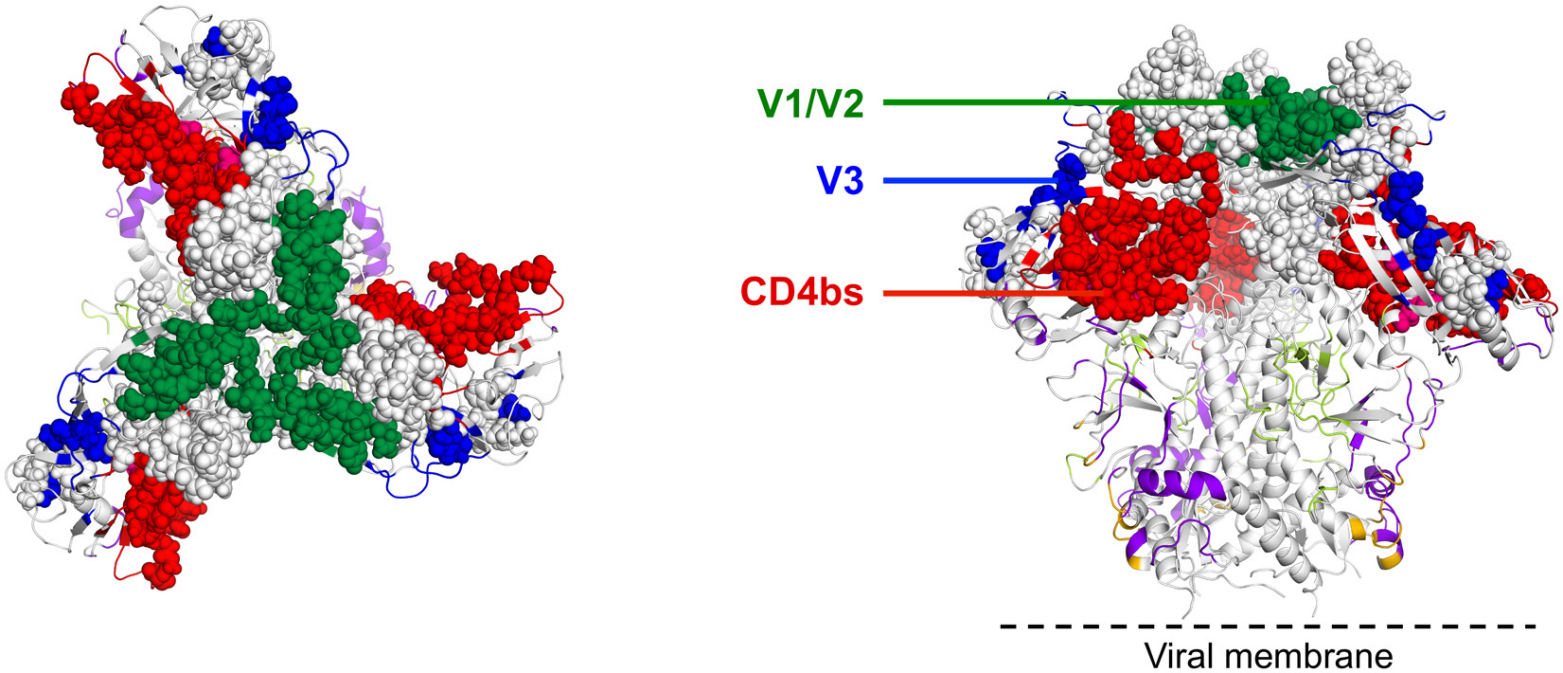
Neutralization epitopes of SIV Env modeled onto the 3D structure of HIV-1 BG505 SOSIP.664 trimer. Pre-fusion HIV-1 BG505 trimer structure (PDB ID: 4TVP) is displayed in ribbon representation with red for CD4-binding-site-directed antibody specificities (VRC01-, b12-, CD4-, and HJ16-like), purple for 8ANC195-like, green for V1V2-directed (PG9-like), blue for glycan-V3 specificities (PGT128- and 2G12-like), orange for 35O22-like specificities, and green-yellow for PGT151-like specificities. Epitope targets of SIV Env neutralizing mAbs are shown as spheres with red for CD4-binding-site-, green for V1V2-, and blue for V3-directed specificities. Top (left) and side (right) views are shown.

Overall, we isolated multiple SIV mAbs directed against major targets of SIV Env vulnerability analogous to bnAb targets of HIV-1, namely the CD4bs, peptide epitopes of V1/V2 and V3 loops and potentially glycan targets of SIV Env (Fig 14). We did not isolate SIV mAbs targeting the V4 loop although it is possible that some of the SIV mAbs from the CVNbs sort may map to this region. Targeted isolation of V4-specific SIV mAbs could prove useful as the V4 region of SIV contains immunodominant epitopes and represents an early target for neutralizing mAbs [108, 109]. Compared with HIV-1 bnAb targets, we were unable to isolate quaternary-structure-preferring SIV-specific mAbs. While a pre-fusion SIV trimer structure has yet to be determined, it is likely that the SIV foldon trimer and 1JO8-scaffolded probes used for B cell sorting adopt an open quaternary conformation, analogous to HIV-1 soluble, uncleaved trimers [32, 110], which likely precluded us from isolating SIV quaternary-structure-preferring neutralizing mAbs. In fact, structural analysis of vaccine-induced HIV CD4bs-directed mAbs has revealed that despite high affinity binding to soluble Env ligands such as foldon trimers, such mAbs display a suboptimal angle of approach resulting in non-bnAbs with limited breadth and lack of neutralization activity against neutralization-resistant isolates such as JRFL [111]. Additional methods will be needed to isolate SIV bnAbs targeting quaternary epitopes, V1, V2 or V3 glycans, the immunodominant V4 region, the gp120-gp41 interface and MPER region.

The range of SIV mAbs isolated includes binding, non-neutralizing mAbs as well as strain-specific and cross-neutralizing mAbs exhibiting varying degrees of neutralization breadth and potency. We isolated SIV mAbs from SIV-vaccinated, pre-challenge as well as SIV-vaccinated and infected macaques. All but one of the SIV mAb epitopes identified from the latter showed high sequence similarity among SIV isolates tested making it difficult to determine whether individual mAb responses were elicited by the immunogen or the challenge virus Env. Only one of the SIV mAb epitopes identified (Env 142–15) was present in the SIVsmE660 challenge virus but not the SIVmac239 immunogen indicating that mAbs targeting this region were elicited following infection.

Comparison of the neutralization profiles for individual mAbs and corresponding serum samples for most animals revealed that the isolated mAbs recapitulated the breadth of serum neutralization in most cases (S9 Fig). In addition, the neutralization plateau effect reported for some HIV-1-specific mAbs [16] was also evident for neutralization curves of SIV mAbs against tier 2 isolates of both SIVmac251 and SIVsmE660, irrespective of mAb specificity. While differences in Env trimer glycosylation may explain incomplete neutralization by glycan-dependent mAbs [112], emerging data suggests conformational heterogeneity of Env trimers even within a clonal pseudovirus population may account for neutralization curve plateaus for glycan-independent mAbs [38, 113]. Other groups have also observed striking heterogeneity in neutralization sensitivities between SIV isolates [78, 82]. While the majority of clones within the well-characterized SIVsmE660 vaccine challenge stock are highly neutralization sensitive, approximately 10–25% exhibit an intermediate neutralization sensitivity phenotype and 10% are outright neutralization resistant [82]. Despite the broad range of epitopes targeted by SIV mAbs isolated and their capacity to bind SIVmac239 gp140 FT protein, none were able to neutralize the highly neutralization resistant SIVmac239. This was not wholly unexpected since sera from these animals also failed to neutralize SIVmac239 (Fig 12). This discrepancy between binding and neutralizing activity against a particular Env protein/virus has also been observed for HIV-1 CD4bs-directed mAbs and is thought to be result from inefficient recognition of cognate epitope due to quaternary packing conformational constraints in the context of functional, membrane-bound trimer despite high affinity binding to soluble Env ligands [111, 114, 115].

With the development of our novel competitive probe binding sort strategy and subsequent isolation and detailed characterization of nearly 70 SIV Env-specific mAbs we now have the necessary reagents with which to study immune and vaccine mediated correlates of protection in the SIV NHP challenge model of HIV-1. This includes testing of SIV bnAb passive immunization alone or as an adjunct to antiretroviral therapy (ART) by direct injection or gene therapy. As well, the binding, non-neutralizing SIV Env-specific mAbs identified here will serve as useful reagents for delineating the contribution of antibody-dependent cellular cytotoxicity (ADCC), antibody-dependent cell-mediated viral inhibition (ADCVI) and additional FcR-mediated activities toward control and/or prevention of HIV/SIV infection. Finally, use of additional probes and methods to isolate SIV-specific B cells will facilitate more thorough and rigorous pre-clinical evaluation of mAb-based immunotherapies for treatment and/or prevention of SIV infection in NHPs.

## Supporting Information

S1 Table Summary of vaccination and infection history of rhesus macaque PBMC/serum samples. (TIF)

S2 Table Primer sequence pools for amplifying rhesus IgG V-regions. (TIF)

S3 Table Primer sequence pools for amplifying rhesus IgLκ V-regions. (TIF)

S4 Table Primer sequence pools for amplifying rhesus IgLλ V-regions. (TIF)

S1 Fig Neutralization-resistant SIVmac239. (TIF)

S2 Fig Amino acid sequence alignment of SIV/HIV-2 Env isolates. (TIF)

S3 Fig Amino acid sequence alignment of SIV/HIV-2 Env isolates. (TIF)

S4 Fig Amino acid sequence alignment of SIV/HIV-2 Env isolates. (TIF)

S5 Fig Amino acid sequence alignment of SIV/HIV-2 Env isolates. (TIF)

S6 Fig Neutralization activity of SIV CD4bs mAbs. (TIF)

S7 Fig Neutralization activity of SIV mAbs isolated by CVN competitive binding to SIV gp140. (TIF)

S8 Fig Neutralization activity of SIV V1V2 mAbs. (TIF)

S9 Fig Serum neutralization. (TIF)
