# Correction: Targeted Isolation of Antibodies Directed against Major Sites of SIV Env Vulnerability

**DOI:** 10.1371/journal.ppat.1005674

**Published:** 2016-05-24

**Authors:** Rosemarie D. Mason, Hugh C. Welles, Cameron Adams, Bimal K. Chakrabarti, Jason Gorman, Tongqing Zhou, Richard Nguyen, Sijy O’Dell, Sabrina Lusvarghi, Carole A. Bewley, Hui Li, George M. Shaw, Zizhang Sheng, Lawrence Shapiro, Richard Wyatt, Peter D. Kwong, John R. Mascola, Mario Roederer

Xuejun Chen is not included in the author byline. He should be listed as the seventh author and affiliated with the Vaccine Research Center, National Institute of Allergy and Infectious Diseases (NIAID), National Institutes of Health (NIH), Bethesda, Maryland, United States of America. The contributions of this author are as follows: Performed the experiments.
