# Supplementary figures and images for: Targeted Isolation of Antibodies Directed against Major Sites of SIV Env Vulnerability
Source: PLoS Pathog. 2016 Apr 11;12(4):e1005537. doi: 10.1371/journal.ppat.1005537 (PMC4827850; doi:10.1371/journal.ppat.1005537)

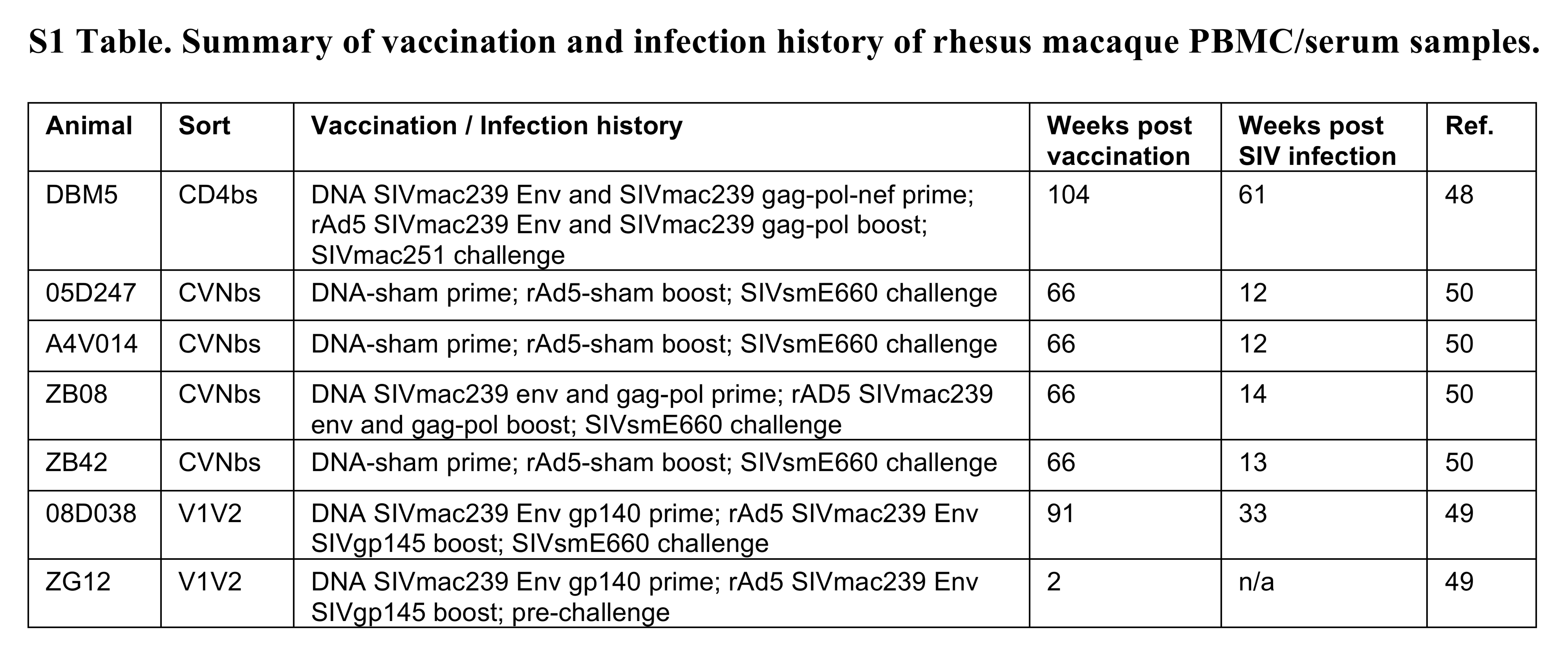

Supplement: S1 Table — (TIF) [file ppat.1005537.s001.tif]

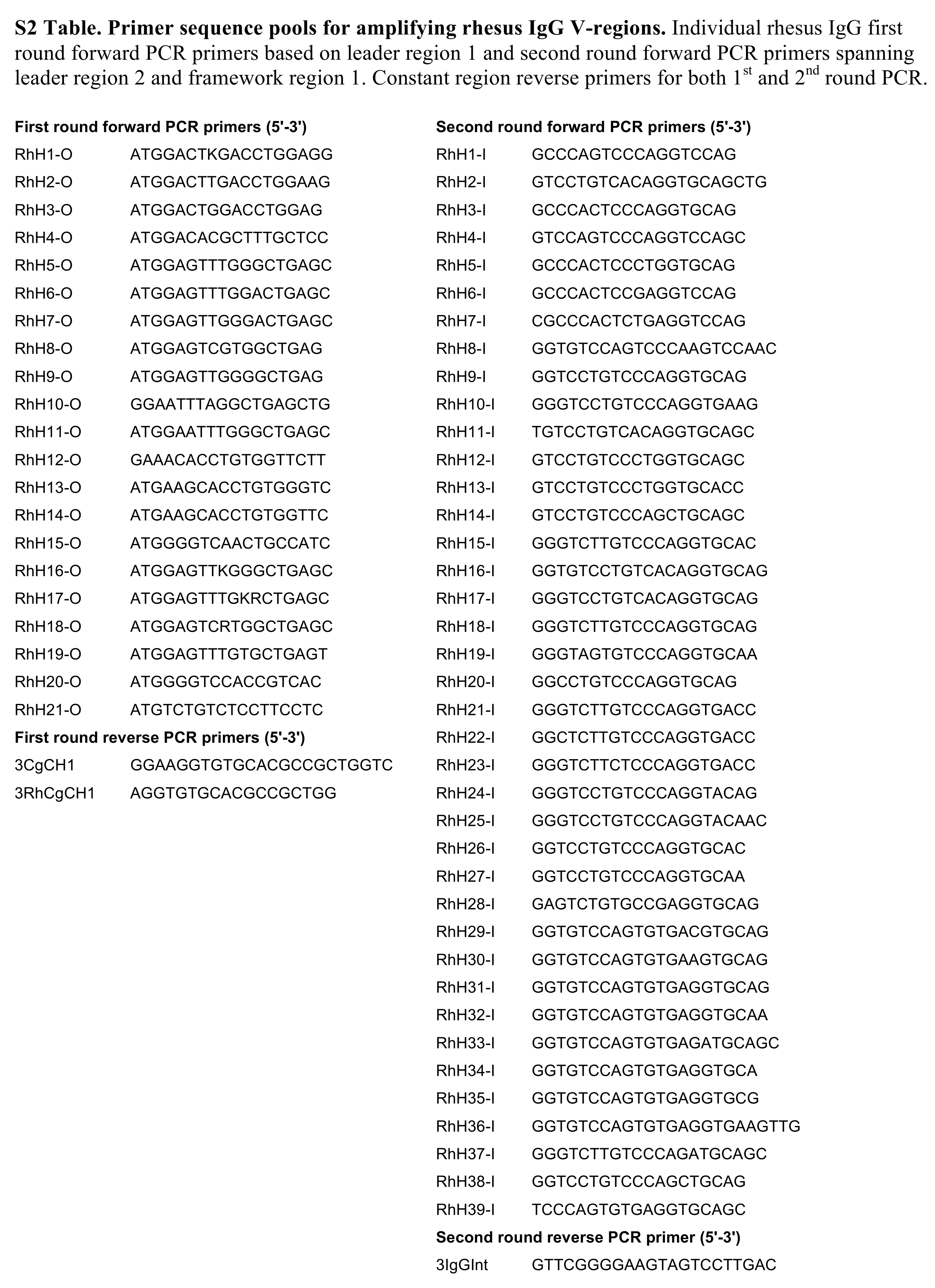

Supplement: S2 Table — (TIF) [file ppat.1005537.s002.tif]

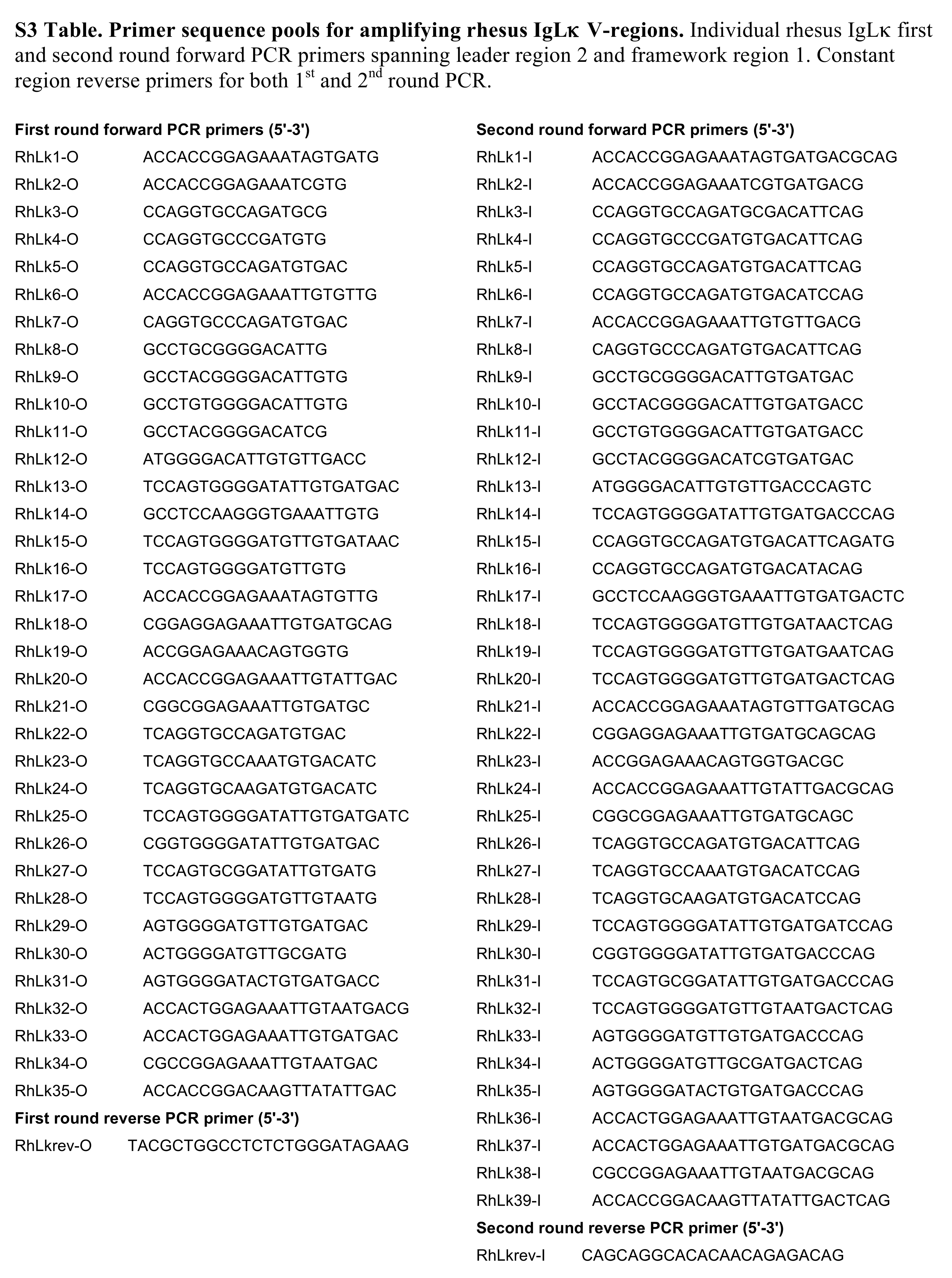

Supplement: S3 Table — (TIF) [file ppat.1005537.s003.tif]

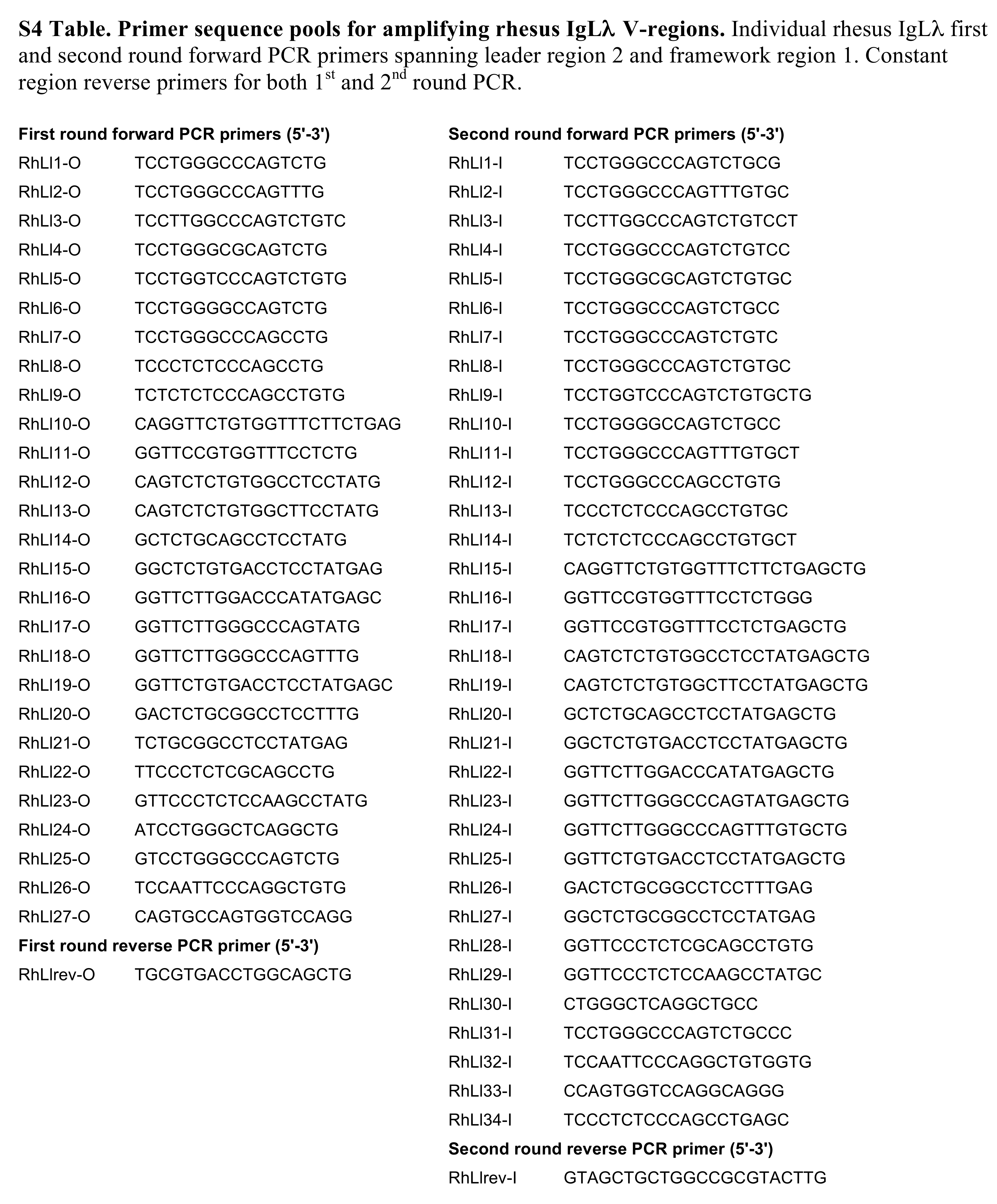

Supplement: S4 Table — (TIF) [file ppat.1005537.s004.tif]

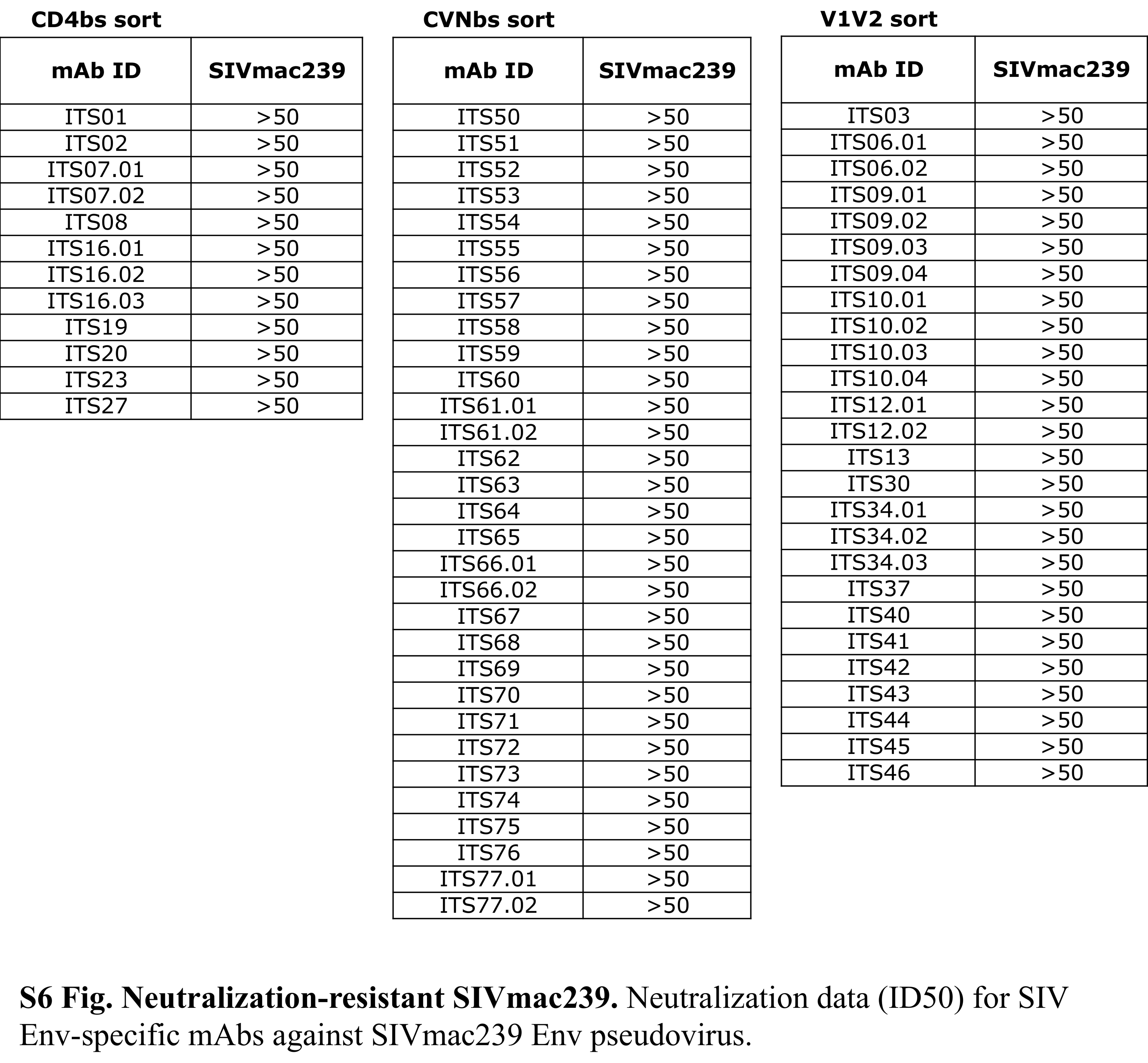

Supplement: S1 Fig — (TIF) [file ppat.1005537.s005.tif]

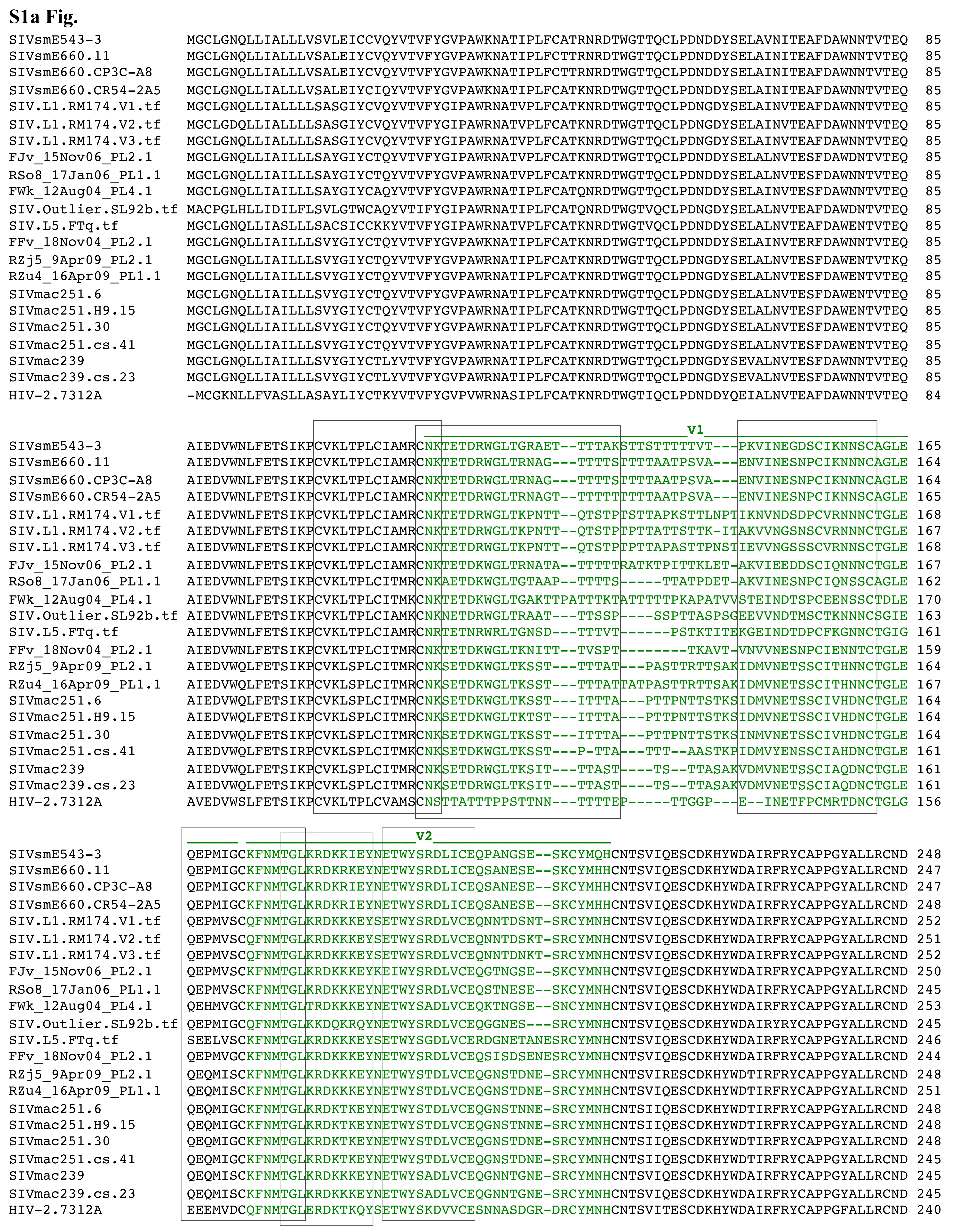

Supplement: S2 Fig — (TIF) [file ppat.1005537.s006.tif]

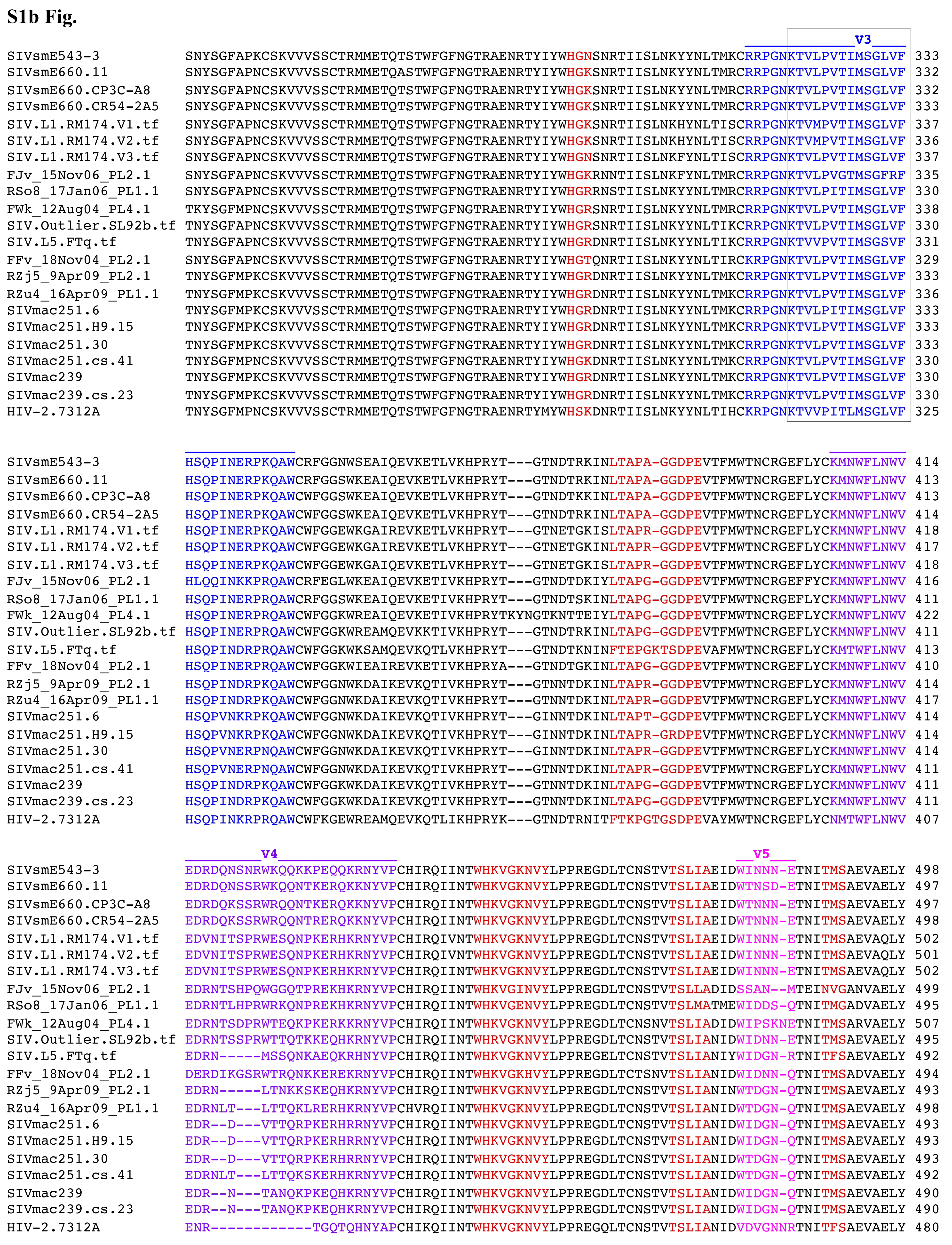

Supplement: S3 Fig — (TIF) [file ppat.1005537.s007.tif]

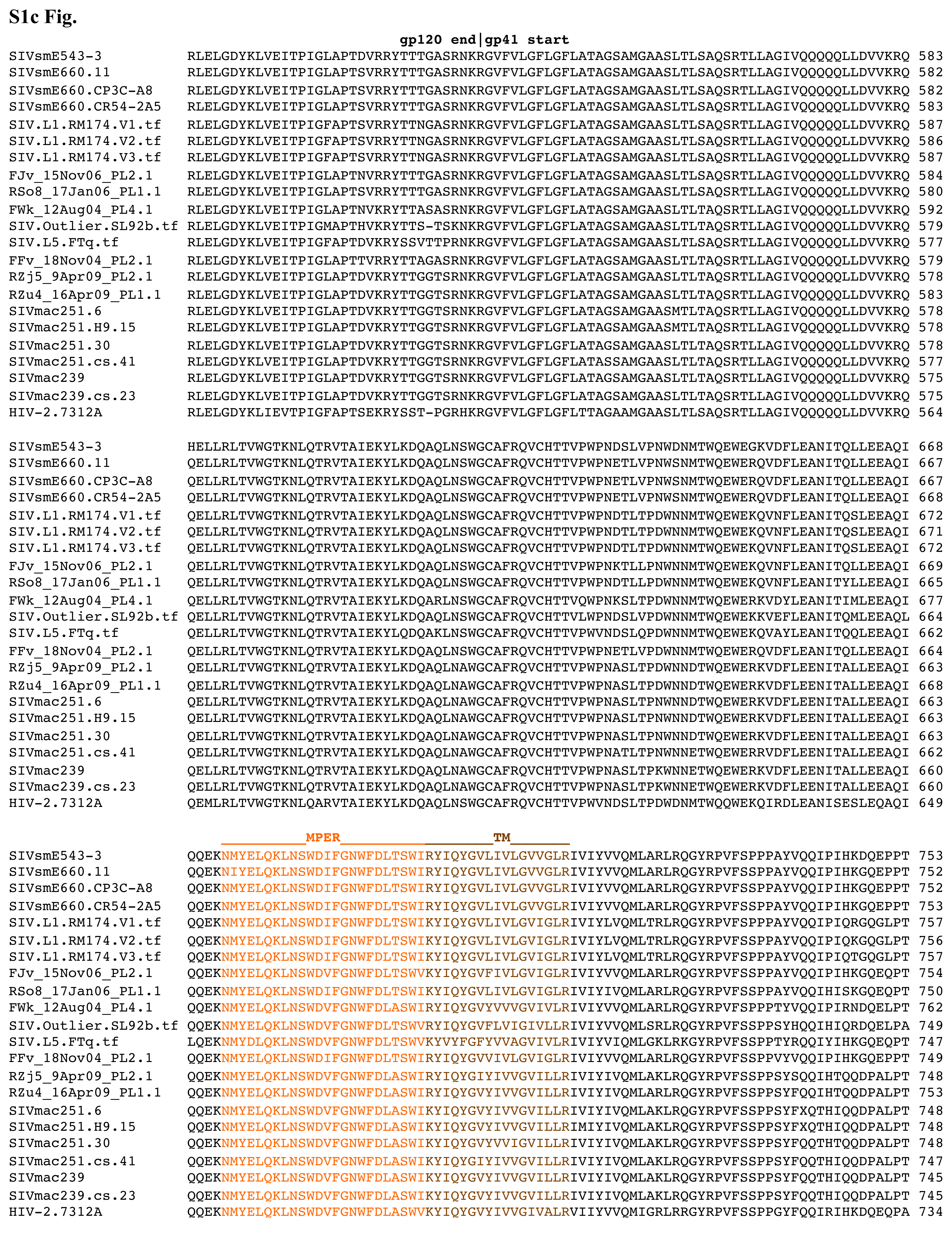

Supplement: S4 Fig — (TIF) [file ppat.1005537.s008.tif]

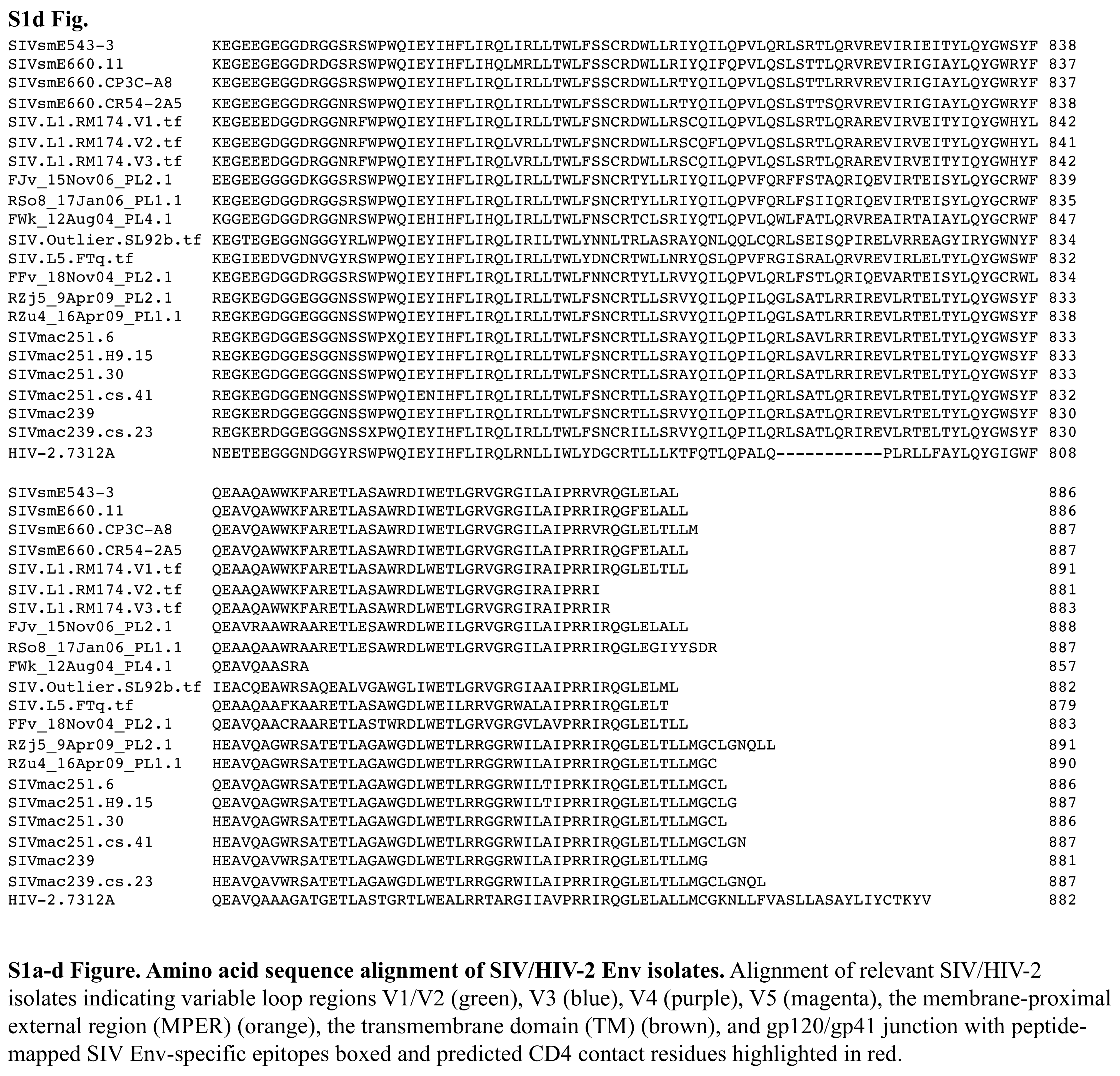

Supplement: S5 Fig — (TIF) [file ppat.1005537.s009.tif]

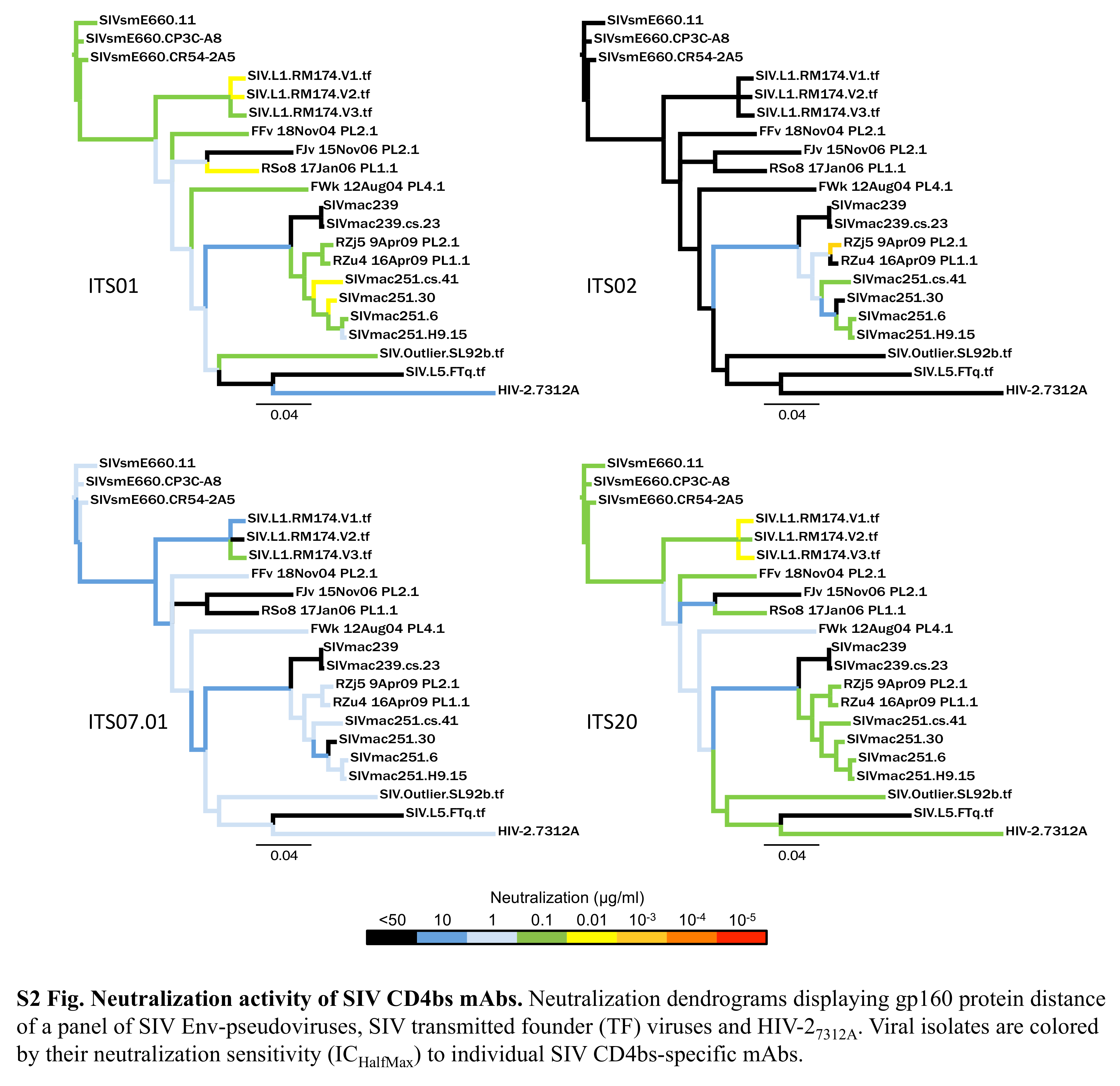

Supplement: S6 Fig — (TIF) [file ppat.1005537.s010.tif]

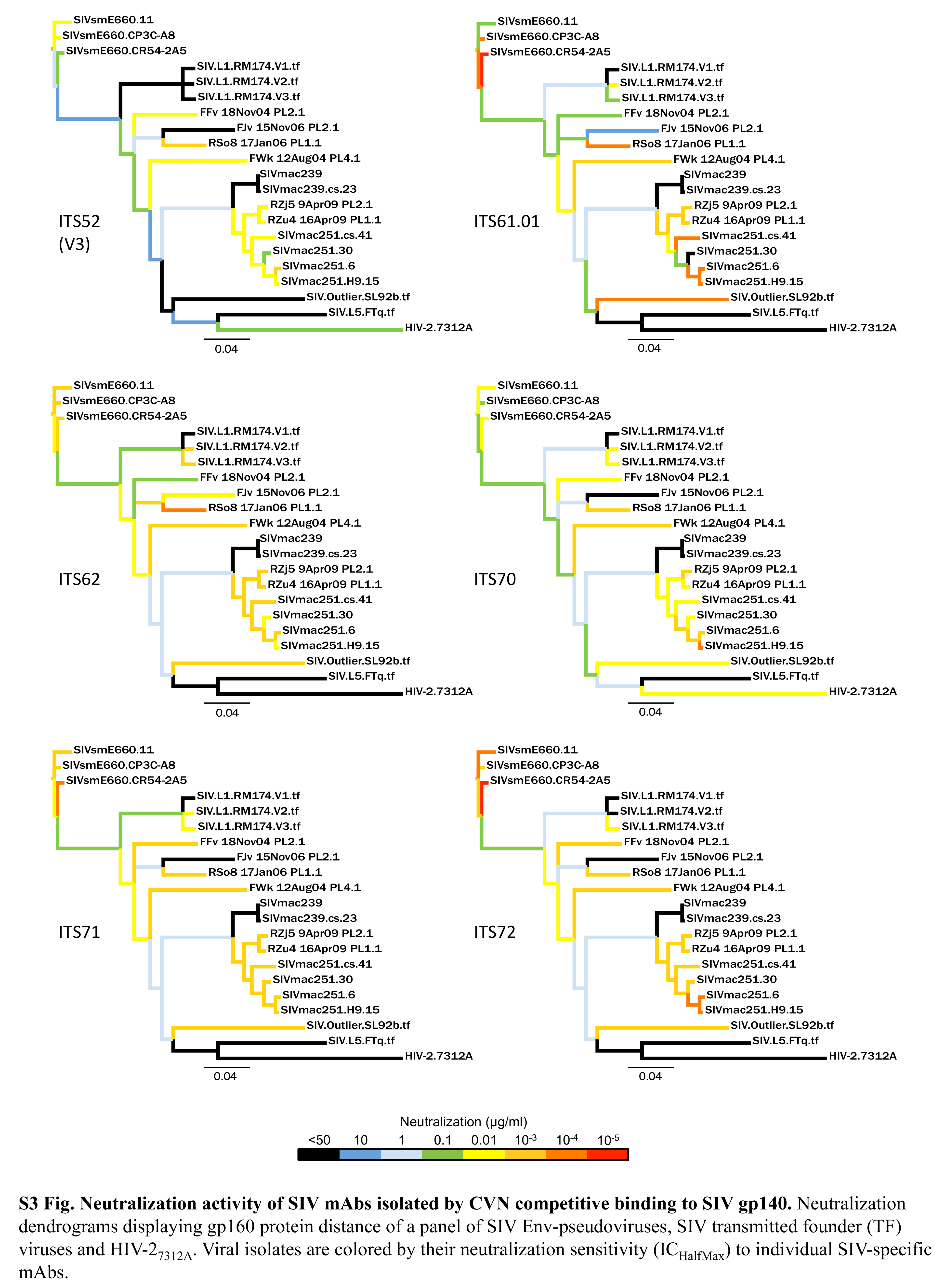

Supplement: S7 Fig — (TIF) [file ppat.1005537.s011.tif]

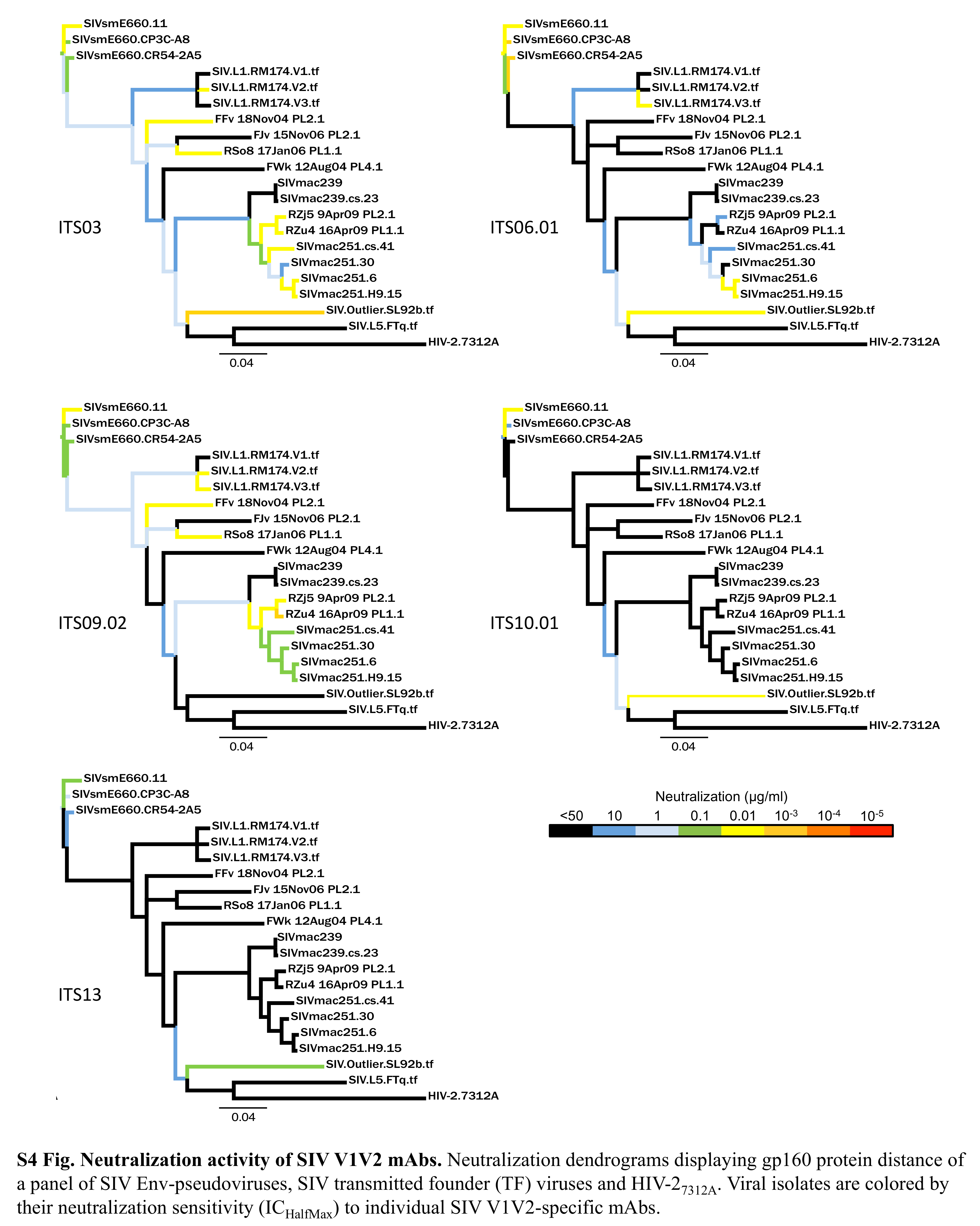

Supplement: S8 Fig — (TIF) [file ppat.1005537.s012.tif]

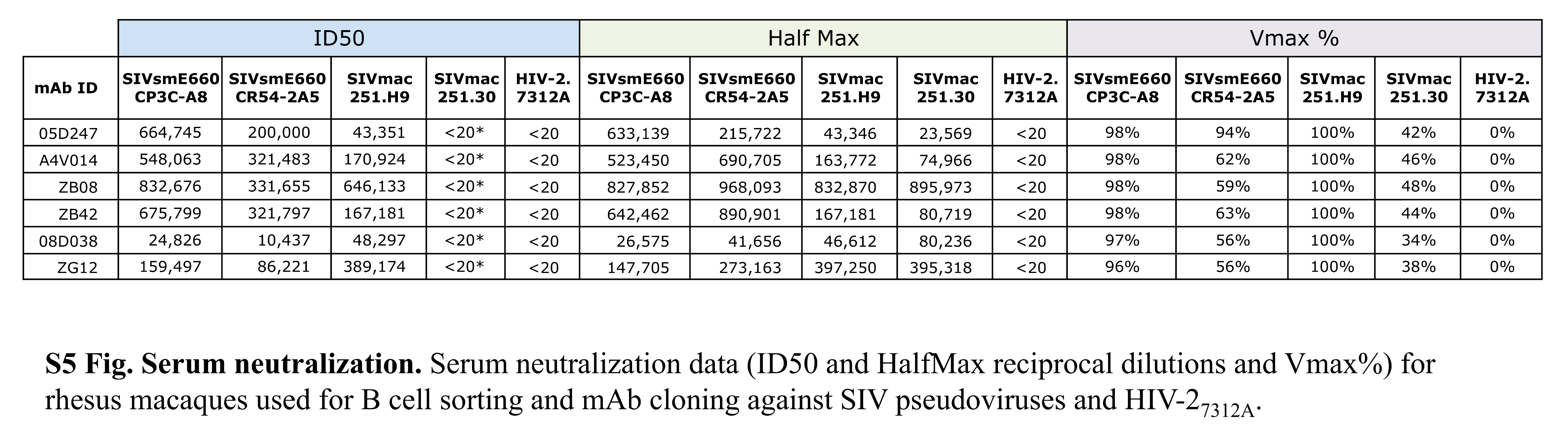

Supplement: S9 Fig — (TIF) [file ppat.1005537.s013.tif]
